# Multicentric Genome-Wide Association Study for Primary Spontaneous Pneumothorax

**DOI:** 10.1371/journal.pone.0156103

**Published:** 2016-05-20

**Authors:** Inês Sousa, Patrícia Abrantes, Vânia Francisco, Gilberto Teixeira, Marta Monteiro, João Neves, Ana Norte, Carlos Robalo Cordeiro, João Moura e Sá, Ernestina Reis, Patrícia Santos, Manuela Oliveira, Susana Sousa, Marta Fradinho, Filipa Malheiro, Luís Negrão, Salvato Feijó, Sofia A. Oliveira

**Affiliations:** 1 Instituto de Medicina Molecular, Faculdade de Medicina, Universidade de Lisboa, Lisboa, Portugal; 2 Instituto Gulbenkian de Ciência, Oeiras, Portugal; 3 Hospital Infante Dom Pedro, Aveiro, Portugal; 4 Centro Hospitalar do Porto, Porto, Portugal; 5 Centro Hospitalar e Universitário de Coimbra, Coimbra, Portugal; 6 Centro Hospitalar de Vila Nova de Gaia, Vila Nova de Gaia, Portugal; 7 Universidade de Évora, Évora, Portugal; 8 Hospital de São Bernardo (Centro Hospitalar de Setúbal, E.P.E.), Setúbal, Portugal; 9 Hospital Egas Moniz (Centro Hospitalar de Lisboa Ocidental), Lisboa, Portugal; 10 Hospital da Luz, Lisboa, Portugal; 11 Instituto Português do Sangue e da Transplantacão, Centro Regional de Sangue de Lisboa, Lisboa, Portugal; 12 Hospital de Santa Maria, Lisboa, Portugal; NIH - National Institute of Environmental Health Sciences, UNITED STATES

## Abstract

Despite elevated incidence and recurrence rates for Primary Spontaneous Pneumothorax (PSP), little is known about its etiology, and the genetics of idiopathic PSP remains unexplored. To identify genetic variants contributing to sporadic PSP risk, we conducted the first PSP genome-wide association study. Two replicate pools of 92 Portuguese PSP cases and of 129 age- and sex-matched controls were allelotyped in triplicate on the Affymetrix Human SNP Array 6.0 arrays. Markers passing quality control were ranked by relative allele score difference between cases and controls (|RAS_diff_|), by a novel cluster method and by a combined Z-test. 101 single nucleotide polymorphisms (SNPs) were selected using these three approaches for technical validation by individual genotyping in the discovery dataset. 87 out of 94 successfully tested SNPs were nominally associated in the discovery dataset. Replication of the 87 technically validated SNPs was then carried out in an independent replication dataset of 100 Portuguese cases and 425 controls. The intergenic rs4733649 SNP in chromosome 8 (between *LINC00824* and *LINC00977*) was associated with PSP in the discovery (*P* = 4.07E-03, OR_C_[95% CI] = 1.88[1.22–2.89]), replication (*P* = 1.50E-02, OR_C_[95% CI] = 1.50[1.08–2.09]) and combined datasets (*P* = 8.61E-05, OR_C_[95% CI] = 1.65[1.29–2.13]). This study identified for the first time one genetic risk factor for sporadic PSP, but future studies are warranted to further confirm this finding in other populations and uncover its functional role in PSP pathogenesis.

## Introduction

Primary Spontaneous Pneumothorax (PSP) is characterized by presence of air in the pleural cavity without preceding trauma or known cause. The annual incidence of PSP is 1–28 cases per 100,000 individuals, typically affecting tall, thin, smoking young males [1–5]. This is the only condition in which young patients are discharged after a first episode having a very high probability of recurrence [3–5] and no effective secondary prevention measures. Available therapeutic options vary from simple aspiration with a catheter to complex video-assisted thoracoscopic surgery, but optimal management of PSP remains controversial [3].

Approximately 10% of PSP patients have a positive family history [6], with an autosomal dominant inheritance with incomplete penetrance, X-linked recessive or polygenic inheritance [6–8]. Mutations in the folliculin gene have been identified in individuals with familial PSP [8–12], but the genetic aetiology of sporadic PSP remains unknown since there are no published genetic association studies.

Genome-wide association study (GWAS) is a highly-powered association strategy covering the entire genome with densely distributed markers, looking for common variants that contribute to complex traits risk in an unbiased way. GWAS marked a new era in the complex disorders field and led to the discovery of several causal *loci*. These studies are powerful but remain very expensive and time-consuming. Thus, DNA-pooling strategies combined with microarray genotyping have proven effective in reducing costs and in identifying risk loci in several studies [13–19]. Here we used this validated strategy to perform the first GWAS for PSP. In DNA pooling, genotyping pools of individuals replace individual genotyping. Hence, equimolar amounts of DNA are combined from each sample to form cases and controls pools, to assess any allelic frequency differences between these. Subsequently, only a fraction of the most significant SNPs will be validated using individual genotyping groups [13]. Using different high-density genotyping platforms and numerous analysis methods to rank the polymorphisms, this pooling GWAS strategy has been validated by the replication of known associations while identifying new *loci* for other complex disorders [20–22]. We thus applied this strategy to the field of PSP genetics to pioneer the search for genes involved in its susceptibility, using a southern European population.

## Materials and Methods

### Participants

All study participants are Portuguese of Southern European descent. Patients were diagnosed with idiopathic PSP according to the criteria described in Henry *et al*. [3]. Individuals were excluded if known to have medical disorders etiologically associated with pneumothoraces, namely alpha1-antitrypsin deficiency, Marfan syndrome, Ehlers-Danlos syndrome, Birt-Hogg-Dubé syndrome, cystic fibrosis, histiocytosis, pulmonary lymphangiomyomatosis and sarcoidosis. A thorough medical history was performed for each patient, including information on PSP family history, pulmonary disorders, smoking, physical activity, medication, anthropometric measures, and a detailed characterization of the PSP episodes. If thoracoscopy was performed, macroscopic stages were defined according to Vanderschueren classification as follows: I–normal visceral pleura, II–some pleural adhesions, III–blebs or bullae (emphysema-like changes) <2cm in diameter, and IV–bullae >2cm in diameter [23–25]. Recurrence was defined as a novel pneumothorax episode occurring in more than 1-month period after the end of the treatment in patients that achieved full lung expansion after the first episode.

Pneumologists ascertained patients at Hospital de Santa Maria (Lisboa), Centro de Pneumologia, Faculdade de Medicina da Universidade de Coimbra (Coimbra), Hospital Infante Dom Pedro (Aveiro), Centro Hospitalar de Vila Nova de Gaia (Vila Nova de Gaia), Hospital de São Bernardo (Setúbal), Hospital de Santo António (Porto), Hospital da Luz (Lisboa), and Centro Hospitalar de Lisboa Ocidental (Lisboa). The controls were collected through the Instituto Português do Sangue e da Transplantação (Lisboa) and requested from Biobanco-IMM, Lisbon Academic Medical Center, Lisbon, Portugal. The ethical committee of Hospital de Santa Maria approved this study and all participants provided written informed consent.

### Construction of DNA pools

DNA was extracted as described previously [26]. Quantification of genomic DNA was performed in triplicate using the Picogreen^®^dsDNA Quantitation Kit (Invitrogen, Oregon, USA) in a PerkinElmer top Fluoroscence reader (PerkinElmer, Inc., Waltham, USA). Samples with values >3% of the sample standard deviation (SD) between replicates or with values >2 SDs from the median volume to be pooled for each sample (2 PSP cases and 6 controls) were not included in the respective pool. DNA (200ng) from each sample that passed quality control (92 cases and 129 controls) was then added to either a case or a control pool. Each pool was assembled, quantified and adjusted to 50ng/μl. To minimize pipetting-associated errors, no less than 1.7uL of each sample was added to a pool. This procedure was repeated twice so that two replicate pools of cases and of controls were constructed.

### Genomewide allelotyping

High-throughput allelotyping of 906,000 SNPs was performed in triplicate on Affymetrix Human SNP Array 6.0 (Santa Clara, California, USA) at Instituto Gulbenkian de Ciência’s Microarray Core Facility using standard protocols. After thorough quality control, probe intensity data was transferred to the R statistical platform (http://www.r-project.org) and normalized across chips using the SNPMaP package [27]. SNPMaP identified and removed 38,338 SNPs performing poorly (e.g. located in sex chromosomes, CNV regions and mitochondria), and calculated the Relative Allele Scores (RAS), the pooling equivalent of a relative allele frequencies. RAS usually correspond to the ratio of the A probe to the sum of the A and B probes (where A is the major allele and B is the minor allele). However, with Affymetrix arrays, each SNP is assayed as quartets of perfect match (PM) and mismatch (MM) probes and the RAS score is corrected for the non-specific hybridisation (mismatch probes). The RAS for the sense strand is therefore the median(s_i_^(s)^), where s_i_^(s)^ (median of relative allele signal for the i^th^ probe quartet of the sense strand) is defined as s_i_^(s)^ = A_i_^(s)^ / (A_i_^(s)^ + B_i_^(s)^), given that A_i_^(s)^ = max(PM_i_^(sA)^–MM_i_^(s)^, 0), B_i_^(s)^ = max(PM_i_^(sB)^–MM_i_^(s)^, 0), and the average mismatch signal is MM_i_^(s)^ = (MM_i_^(sA)^ + MM_i_^(sB)^)/2 [28]. Twelve RAS values were calculated for each SNP (from six case and six control pools) and used for the subsequent analysis. All markers in the mitochondrial genome were also excluded given that no normalization for mitochondrial DNA copy number was performed at the pooling stage. A Pearson’s correlation coefficient was calculated between the average of the RAS values of cases and controls using R. The modified Manhattan plot was also built using R.

### Cluster method

SNPs with an absolute value of the RAS difference (|RAS_diff_|) ≥8% were annotated according to the University of California at Santa Cruz (UCSC, GRCh37/hg19, http://genome.ucsc.edu/cgi-bin/hgGateway) and the Affymetrix GenomeWideSNP_6.na32.annot databases and assigned to either a genic cluster or an intergenic cluster. The linkage disequilibrium (LD) between each pair of SNPs within a cluster was calculated from HapMap3 data (http://hapmap.ncbi.nlm.nih.gov/, CEU samples).

### Individual genotyping

Genotyping was performed as described previously [29] using the primer sequences listed in the S1 Table. Deviation (*P*<1.00E-03) of genotype distribution from Hardy-Weinberg equilibrium (HWE) was tested for each marker in the control dataset using Haploview 4.2 [30]. In the technical validation stage, five SNPs failed quality control (monomorphic: rs1525833, rs1526483, and rs2919427; out of HWE: rs2971955 and rs10504160). In the replication phase, three SNPs had a very low call rate (rs230833, rs10508279) or were not in HWE (rs922799). Pairwise LD (r^2^) was calculated and plotted using SNAP (http://www.broadinstitute.org/mpg/snap/ldsearch.php). Haplotype tagging SNPs (htSNPs) in *SLC6A1* (chr.3: 11,009,456–11,055,934 kb) were identified with Tagger from Haploview 4.2 using genotypes of 30 European (HapMap CEU–Utah residents with ancestry from northern and western Europe) family trios (V.3, release 27) and with the following options: aggressive tagging mode, r^2^>0.75 and minimum minor allele frequency (MAF) of 0.05.

### Association analyses

Unpaired Student’s t tests and χ^2^ tests were used to compare quantitative and qualitative clinical and demographic data, respectively, between PSP patients and controls. Association analyses were performed using a logistic regression (linear regression for a dichotomous response variable, in this case affected or unaffected) implemented with the glm function in R. The general equation of the model used is ln[*p*/(1−*p*)] = β_0_ + β_1_X_1_, in which *p* is the probability of being affected, X_1_ is the exploratory variable (assuming values 0, 1, or 2 in the log-additive model depending on the number of reference alleles an individual has at the SNP being investigated), β_0_ is the regression coefficient in the reference group, and β_1_ is the regression coefficient associated with the reference group and the X_1_ explanatory variable [31]. Odds ratios (OR) and 95% confidence intervals (CI) were calculated using β_1_ and its standard error to determine the relative disease risk conferred by a particular allele.

## Results

### DNA pooling and GWAS

The main demographic and clinical characteristics of the discovery dataset used in the GWAS are summarized in Table 1. These 135 controls and 94 PSP cases were matched for age, gender, and mean height (*P* = 8.29E-01, *P* = 7.76E-01, and *P* = 5.87E-02, respectively) and therefore association tests do not need to be adjusted for these three known PSP risk factors, but not for weight, BMI and Rohrer’s index (*P* = 2.91E-09, *P* = 1.99E-16, and *P* = 3.78E-17, respectively). As described previously for spontaneous pneumothoraces, the vast majority of patients were resting at PSP onset (83.3% versus 87% in [32]) when they suddenly felt chest pain (97.8%) [33], dyspnea (81.7%), and cough (34.9%). Almost all patients had unilateral pneumothoraces and approximately 40% of them had recurrent events.

**Table 1:** Main clinical and demographic characteristics of the PSP case-control discovery and replication datasets.

Characteristic	Discovery dataset		Replication dataset	
	Cases	Controls	Cases	Controls
**N**	94	135	100	425
**Gender ratio (M/F)**	6.8:1	6.1:1	3.3:1	4.2:1
**Mean age at examination (years±SD) (n/N)**	26.4±5.2 (94/94)	26.2±5.4 (135/135)	27.5±6.6 (100/100)	23.3±4.5 (425/425)
**Mean height (cm±SD) (n/N)**	177.2±7.7 (94/94)	175.2±7.8 (135/135)	175.3±8.1 (100/100)	174.5±10.0 (425/425)
**Mean weight (kg±SD) (n/N)**	66.2±8.4 (94/94)	75.4±12.6 (135/135)	66.2±10.6 (100/100)	72.4±12.2 (425/425)
**BMI (kg/m2±SD) (n/N)**	21.0±2.0 (94/94)	24.5±3.3 (135/135)	21.5±2.9 (100/100)	23.6±3.23 (425/425)
**Rohrer’s index (kg/m3±SD) (n/N)**	118.9±12.8 (94/94)	139.9±19.5 (135/135)	122.8±17.3 (100/100)	135.3±18.8 (425/425)
**Symptoms at onset**				
Cough (%) (n/N)	34.9 (30/86)	-	30.9 (30/97)	-
Dyspnea (%) (n/N)	81.7 (76/93)	-	65.0 (63/97)	-
Chest pain (%) (n/N)	97.8 (91/93)	-	94.0 (94/100)	-
**Onset**				
During physical activity (%) (n/N)	16.7 (15/90)	-	26.4 (24/91)	-
At rest (%) (n/N)	83.3 (75/90)	-	73.6 (67/91)	-
**Recurrence (%) (n/N)**	40.4 (38/94)	-	29.0 (29/100)	-
**1st PSP EPISODE**				
**N**	94		100	
**Mean age (years±SD) (n/N)**	25.3±5.4 (92/92)	-	26.4±7.0 (100/100)	-
**Affected lung**				
Left (%) (n/N)	47.8 (43/90)		51.0 (51/100)	
Right (%) (n/N)	51.1 (46/90)		49.0 (49/100)	
Bilateral (%) (n/N)	1.1 (1/90)		0.0 (0/100)	
**Mean collapse (%±SD) (n/N)**	45.6±24.3 (55/55)	-	51.5±28.3 (71/71)	
Macroscopic stage^*^ **(%) (n/N)**	I—18.9 (7/37)		I—15.8 (3/19)	
	II—40.5 (15/37)		II—26.3 (5/19)	
	III—29.7 (11/37)		III—47.4 (9/19)	
	IV—10.8 (4/37)		IV—10.5 (2/19)	
**2nd PSP EPISODE**				
**N**	38		34	
**Mean age (years±SD) (n/N)**	24.3±4.7 (38/38)	-	23.8±5.7 (34/34)	
**Affected lung**				
Left (%) (n/N)	51.4 (19/38)		58.8 (20/34)	
Right (%) (n/N)	43.2 (16/38)		38.2 (13/34)	
Bilateral (%) (n/N)	5.4 (2/38)		2.9 (1/34)	
**Mean collapse (%±SD) (n/N)**	44.3±21.1 (18/18)		38.7±23.8 (23/23)	
Macroscopic stage^*^ **(%) (n/N)**	I—8.3 (1/12)		I—0.0 (0/6)	
	II—25.0 (3/12)		II—50.0 (3/6)	
	III—66.7 (8/12)		III—33.3 (2/6)	
	IV—0.0 (0/12)		IV—16.7 (1/6)	
**3rd PSP EPISODE**				
**N**	13		12	
**Mean age (years±SD) (n/N)**	23.2±4.0 (13/13)		23.6±5.1 (12/12)	
**Affected lung**				
Left (%) (n/N)	25.0 (3/12)		54.6 (6/11)	
Right (%) (n/N)	66.7 (8/12)		45.4 (5/11)	
Bilateral (%) (n/N)	8.3 (1/12)		0.0 (0/11)	
**Mean collapse (%±SD) (n/N)**	30.8±31.2 (6/6)		36.5±23.0 (8/8)	
Macroscopic stage^*^ **(%) (n/N)**	I—50.0 (1/2)		I—50.0 (1/2)	
	II—50.0 (1/2)		II—50.0 (1/2)	
	III—0.0 (0/2)		III—0.0 (0/2)	
	IV—0.0 (0/2)		IV—0.0 (0/2)	

Abbreviation: SD, standard deviation.

*Macroscopic stages were defined according to Vanderschueren classification [23–25] as follows: I–normal visceral pleura, II–some pleural adhesions, III–blebs or bullae (emphysema-like changes) <2cm in diameter, and IV–bullae >2cm in diameter.

DNA samples from these study participants met our quality controls and were therefore pooled in equimolar amounts in duplicate and allelotyped in triplicate on Affymetrix Genome-Wide Human SNP Array 6.0 assaying 906 600 SNPs (total of 12 arrays). This strategy was preferred over constructing several smaller pools and hybridizing them on single arrays as most of the pooling error is due to variation between arrays, not to variation in pool construction [15, 34]. Still, the added variance created by pooling specific errors was additionally taken into account in the analysis performed using the combined Z-test [13]. Furthermore, the average of the RAS values over the six case and six control arrays showed a strong Pearson correlation with each other (r = 0.998, S1 Fig), suggesting a low technical variability of the pooling method.

### SNPs prioritization

A total of 868,260 SNPs passed quality controls and were further analyzed. Since there is no single gold-standard method to rank SNPs from GWAS performed on pools, we used three complementary approaches: 1) |RAS_diff_|; 2) cluster method; 3) combined Z-test.

**Fig 1:**
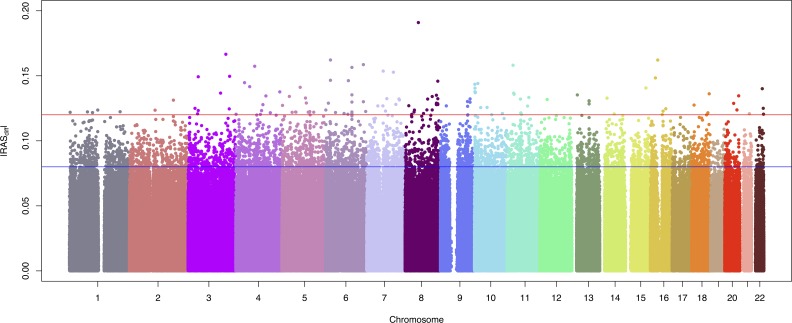
Modified Manhattan plot (|RAS_diff_| against chromosomal location) for the primary spontaneous pneumothorax genome-wide association study. The absolute value of the relative allele score (RAS) difference between cases and controls (|RAS_diff_|) is shown for 868,260 autosomal SNPs allelotyped in 92 PSP patients and 129 healthy controls, ordered by chromosomal position. The red and blue lines represent the 12% and 8% |RAS_diff_| thresholds, respectively.

The absolute value of the |RAS_diff_| in pooling experiments is thought to be a good proxy for allelic frequency difference in individually genotyped datasets [20, 29, 35, 36]. Fig 1 depicts a modified version of a Manhattan plot where |RAS_diff_| between cases and controls is plotted against the genomic position of all the genetic markers passing quality controls. 4589 SNPs had a |RAS_diff_| above background levels (>8%), ranging up to 19% for rs10504160 in chromosome 8. In Fig 1, the density of dots is much higher for |RAS_diff_| below 8%, supporting our choice of 8% for the “background” level cutoff. Another drop in dot density occurs at 12%, with only 135 SNPs with |RAS_diff_|≥12% (listed in S2 Table).

Considering that multiple independent evidences of association pointing to a specific gene or genomic region may be a stronger indication of a true association signal than isolated peaks of association in GWAS with very high SNP density, we devised a cluster approach that highlights weaker but consistent signals of association. SNPs with a |RAS_diff_| above background were assigned to either a gene cluster (if it lies within a gene or up to 10% of its gene size upstream or downstream) or an intergenic cluster (sliding window of 100Kb without overlap containing at least five SNPs), using UCSC and Affymetrix databases. The pairwise LD between each pair of SNPs within a cluster was calculated and an LD score was attributed to each cluster according to the number of independent LD signals within that cluster. Every set of at least two SNPs within one cluster with pairwise LDs (as measured by r^2^) ≥0.8 contribute 1 point to the LD score as they represent a single LD signal. Using this approach, among the 4589 SNPs with a |RAS_diff_|>8%, 1960 SNPs were grouped in 1078 gene clusters and the 2629 SNPs were grouped in 61 intergenic clusters. The clusters with LD score≥5 are listed in S3 Table.

The combined Z-test developed by Abraham *et al*. [20] merges chi-square estimation to assess allelic proportional differences in patients and controls and a Z-statistic for testing mean allelic frequency differences between the same groups. Hence, this approach takes into account both experimental and sampling errors. The 100 SNPs with lower *P*-values according to this test are listed in S4 Table.

### Technical validation

To validate the pool construction, SNPs prioritized using the three above mentioned approaches were selected for technical validation by individual genotyping in the discovery dataset. S5 Table lists the 101 SNPs which were taken into the technical validation phase: 48 SNPs with higher |RAS_diff_| (S2 Table), one SNP per cluster with LD score ≥ 5 (within each cluster, SNPs that had already been selected by the |RAS_diff_| method were selected first, followed by the SNP with highest |RAS_diff_| and MAF>0.05 for which genotyping primers could be designed, S3 Table), and the top 49 SNPs from the combined Z-test (S4 Table). Among these 101 SNPs, seven were selected by all three methods, two markers were convergent between the |RAS_diff_| and cluster methods, twenty-nine markers were convergent between the |RAS_diff_| and combined Z-test approaches, and one SNP was convergent between the cluster and combined Z-test strategies (S5 Table and S2 Fig).

Out of the 101 SNPs selected for individual genotyping in the discovery dataset using the Sequenom technology, five markers failed quality control and the association of another two markers (rs4377469, and rs17133680) could not be assessed using a logistic regression due to the lack of individuals with the rare homozygous genotype. 87 out of the 94 successfully analyzed SNPs (92.6%) were technically validated since they were associated with PSP at the conventional *P*-value of 5.00E-02 (S6 Table).

### Independent replication of GWAS-associated SNPs

For the 87 SNPs that were technically validated, the next step was to assess their association in an independent replication dataset composed of an additional 100 PSP cases and 425 controls (Table 1) matched for gender and height (*P* = 4.10E-01 and *P* = 4.50E-01, respectively), but not for age at examination (*P* = 1.41E-13). A combined analysis was performed using both the discovery and replication datasets (total of 746 individuals) for SNPs that were significantly associated in both the discovery and replication datasets.

Of the 87 genetic markers tested in the replication dataset, three failed quality control (rs230833, rs10508279, and rs922799), and the remaining 84 markers were successfully tested for association in the replication dataset. Among these, the intergenic rs4733649 SNP in chromosome 8 (Table 2) was associated with PSP in the discovery (*P* = 4.07E-03, OR_C_[95% CI] = 1.88[1.22–2.89]), replication (*P* = 1.50E-02, OR_C_[95% CI] = 1.50[1.08–2.09]) and combined datasets (*P* = 8.61E-05, OR_C_[95% CI] = 1.65[1.29–2.13]). Additionally, two SNPs (rs6531429 and 612389) were significantly associated with PSP in both the discovery and replication datasets, but in opposite directions, such that they are not associated in the combined dataset (Table 2).

**Table 2:** PSP association results for the three SNPs associated in the discovery and replication datasets.

SNP	Chr.	Gene (nearest gene)	Allele	Dataset	Case freq.	Control freq.	*P*	OR [95% CI]
rs6531429	4	*LOC439933*	C	Discovery	0.185	0.324	**1.12E-03**	0.45 [0.28–0.73]
				Replication	0.365	0.268	**6.79E-03**	1.58 [1.13–2.19]
				Combined	0.279	0.281	9.32E-01	
rs4733649	8	*LINC00824* (358kb);	C	Discovery	0.429	0.301	**4.07E-03**	1.88 [1.22–2.89]
		*LINC00977* (431kb)		Replication	0.389	0.300	**1.50E-02**	1.50 [1.08–2.09]
				Combined	0.408	0.300	**8.61E-05**	1.65 [1.29–2.13]
rs612389	11	*DLG2*	C	Discovery	0.120	0.213	**1.10E-02**	0.49 [0.28–0.85]
				Replication	0.195	0.118	**3.59E-03**	1.89 [1.23–2.90]
				Combined	0.159	0.140	3.67E-01	

Abbreviations: Chr., chromosome; freq., frequency; *P*, logistic regression *P*-value using the log-additive model.

The SNPs are ordered by chromosomal position. Nominally significant associations are highlighted in bold and their respective OR (odds ratio) and 95% CI (confidence interval) are indicated.

**Fig 2:**
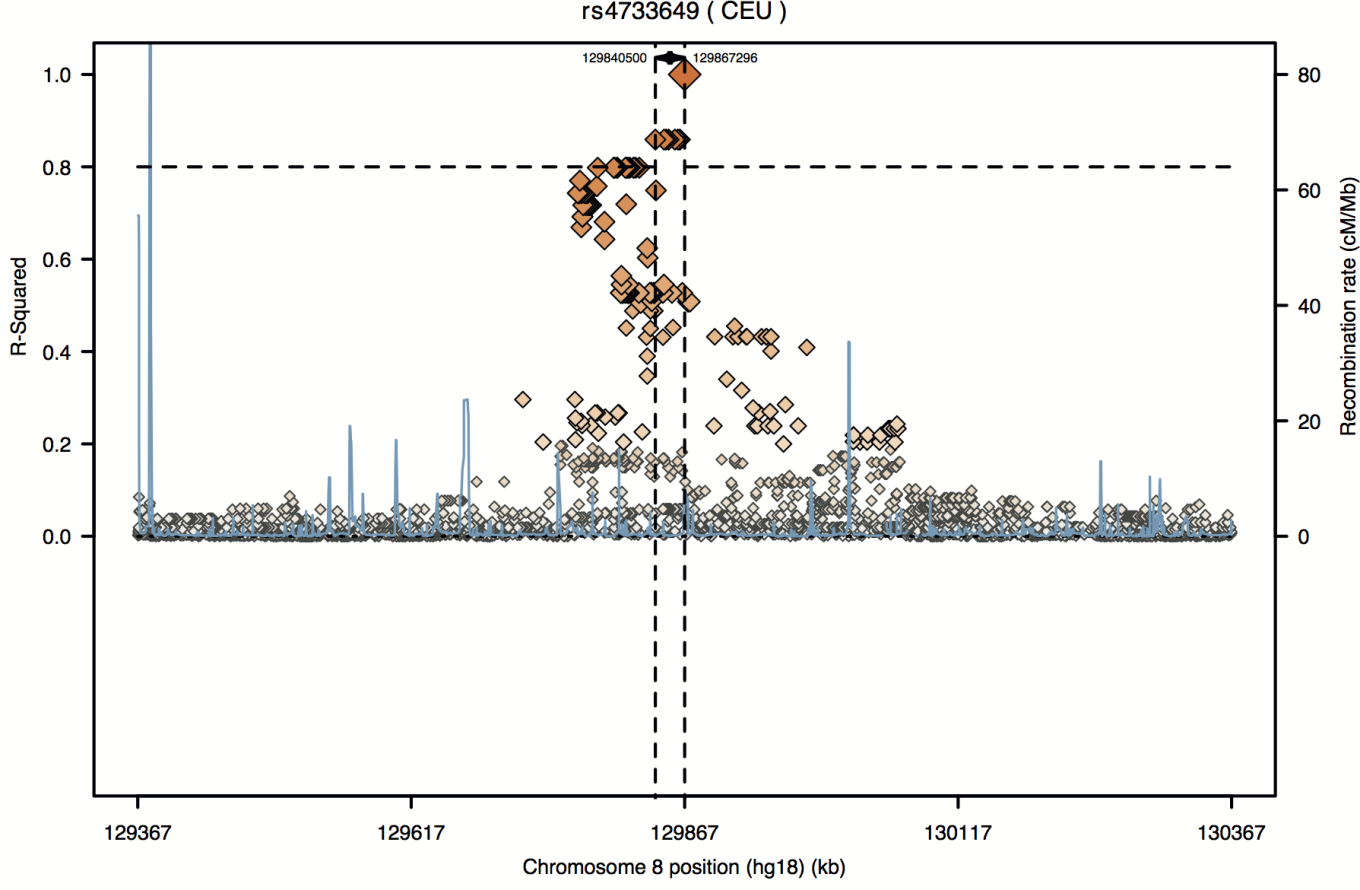
Regional LD plot for rs4733649 at 8q24.21. The pairwise LD (r^2^) between the SNP of interest and surrounding variants and the estimated recombination rate are plotted as a function of genomic position. This plot was constructed by SNAP (SNP Annotation and Proxy Search, http://www.broadinstitute.org/mpg/snap/ldplot.php) using the CEU population panel in the 1000 Genome Project (1000GP) Pilot 1 data and a 500 kilobases (kb) distance limit on each side. The horizontal dashed line is at the 0.8 cut-off for r^2^, and the vertical dashed lines indicate the genomic region encompassing SNPs in strong LD (r^2^≥0.8) with the variant of interest.

To follow-up on the most interesting finding, the regional pattern of LD in the neighboring genomic region of rs4733649 was analysed (Fig 2). Seven neighboring intergenic polymorphisms (rs1519857, rs7460492, rs4545057, rs1367962, rs1432010, rs1432009 and rs2116455) are in strong LD (r^2^≥0.8) with rs4733649 in the CEU population panel of the 1000GP Pilot 1 data (Fig 2).

## Discussion

In this first GWAS ever reported for PSP, the C allele of the intergenic rs4733649 SNP in chromosome 8q24.21 was associated with increased risk for PSP in a discovery, replication and combined datasets of Portuguese PSP cases and controls. The relatively low prevalence of PSP renders the ascertainment and collection of large groups of clinically homogeneous patients an extremely challenging task to carry out and may explain why no association study for PSP has been reported thus far. To increase the statistical power of our study, we used a four-fold larger number of controls than cases in the replication dataset.

To the best of our knowledge, we hereby provide the first comprehensive clinical and demographic characterization of a large PSP dataset. Previous descriptions of datasets have not set apart primary from secondary spontaneous pneumothorax [37] or have focused on specific aspects (e.g. epidemiology, risk factors, management). Some of the characteristics of our datasets are similar to those reported previously (e.g. mean age-at-onset within the 15–34 years range [2], symptoms at onset [32, 33], unilaterality of almost all events [37], 20–60% risk of recurrence [5]), while others differ appreciably (e.g. percentage of PSP cases in each of the macroscopic stages varies widely in different reports [24, 38], probably due the small number of individuals with PSP who undergo thoracoscopy). Curiously, contrary to the common belief that PSP affects taller and thinner individuals [39], the mean height was not significantly different between our cases and controls despite the weight, BMI and Rohrer’s index [39] being significantly different.

There were also some differences between our discovery and replication groups that could, at least in part, explain the non-replication of some initial positive findings. The male to female ratios in our discovery and replication datasets of patients (6.8:1 and 3.3:1, respectively) varied considerably but are similar to reported ratios in the US (6.2:1 in [40]) and England (2.7:1 in [2]), respectively. Even though smoking was more frequent among our PSP cases (59.6% and 72.0% in the discovery and replication datasets, respectively) than among our controls (35.6% and 31.8% in the discovery and replication datasets, respectively) and documented evidence supports a dose-response relationship between smoking and PSP risk [1], we did not include this information in Table 1 or correct for smoking status in the adjusted statistical analyses as data was missing in a number of individuals and was not collected with the desired precision (e.g. quantification, duration).

Since there is no consensus in the literature on which is the most adequate method (e.g. |RAS_diff_|, combined Z-test, F ratio) to prioritize discovery phase results from pool-based GWAS, we used three different approaches. We used the |RAS_diff_| as it seems to be one of the most sensitive methods to pinpoint differences between cases and controls in presence of low technical variation between pools [15, 34], and has previously been successful in identifying risk factors for another complex disease [29]. The biggest disadvantage of this method is that it does not account for RAS variation between replicates, possibly leading to a higher rate of false positives and false negatives, when variations amongst pools are high. To decrease the error attributed to pool construction (biological error) and to array differences (technical error), we prepared two biological replicates and carried out three technical replicates, with a high correlation between arrays. Moreover, we complemented our approach using a combined Z-test [20] that accounts for both experimental and sampling errors, and has proven to be successful in other studies [20]. Furthermore, in parallel, we were inspired by Abraham *et al*. [20] to select SNPs using the cluster method, but decided to include a LD weight, creating an alternative clustering method for both genic and intergenic regions. Technically, the pooling strategy and analysis methods we selected were adequate since the association of 87 out of the 94 successfully tested SNPs was validated by individual genotyping. All three approaches taken were robust in selecting the top findings, since only seven SNPs were not associated in the technical validation phase (five from the LD cluster method and two from the combined Z-test approach). Despite our best efforts, the DNA pooling approach still has limitations and true association signals may have been missed.

Performing multiple statistical tests leads to inflation of false positives, and therefore the statistical significance threshold (usually 5.00E-02) should be adjusted taking into account the number of independent tests. A Bonferroni correction using the total number of SNPs in a GWAS is usually over-conservative given that high linkage disequilibrium between numerous SNPs. The fact that none of the 87 markers tested in the replication dataset would reach the Bonferroni correction threshold (*P*≤5.74E-04) may in part be a consequence of the small sample size and limited power of this study. Furthermore, since this is the very first GWAS ever reported for this disorder, we opted to be more inclusive and less conservative so as not to discard possible interesting findings that must be validated by independent replications in other populations.

rs4733649 maps at 8q24.21, approximately 358 kb and 636 kb downstream from nearest genes *LINC00824* [long intergenic non-protein coding RNA 824] and *MIR1208* [microRNA 1208], respectively, and over 431 kb upstream of *LINC00977* [long intergenic non-protein coding RNA 977]). As observed in most GWAS published to date, the top findings localize to non-coding genomic regions and do not have an immediate functional relevance [41]. Once again, the first and most important step to follow up the association of rs4733649 with PSP is to confirm this finding in a dataset collected by independent researchers. Subsequently, bioinformatics approaches should be pursued to predict the functional consequence of this non-coding variant before designing appropriate molecular experiments [41].

Identification of PSP genetic underpinnings may ultimately have a crucial impact in public health by implementing preventive lifestyle changes in individuals at risk. This study is unique and novel in the pulmonary field, and represents a first step towards controlling PSP.

## Conclusion

In this very first PSP association study, we identified through a comprehensive and unbiased genome-wide approach the first genetic risk factor for sporadic PSP.

## Supporting Information

S1 Fig Scatter plot between average RAS values of cases and controls. Each single nucleotide polymorphisms passing quality control in the pooled-GWAS is represented by a square in a graph where the x- and y-axes indicate the average RAS values of the controls and cases replicates, respectively (plotted using gnuplot 5.0 patchlevel 1 - http://www.gnuplot.info/).
(TIFF)

S2 Fig Venn diagram of the 101 SNPs prioritized for technical validation using the |RAS_diff_|, cluster and combined Z-test methods. The numbers of SNPs chosen by each of the three approaches and overlapping among methods are indicated.
(TIFF)

S1 Table Primer sequences used to genotype the 101 SNPs studied in the technical validation phase. (DOCX)

S2 Table SNPs with |RASdiff|>12% in the PSP GWAS discovery phase. The SNPs are sorted by decreasing |RAS_diff_|, then by chromosomal position, and the top 48 markers highlighted in bold were selected for the technical validation stage.
(DOCX)

S3 Table SNP composition of the 54 genic and intergenic clusters with LD score ≥ 5 in the PSP GWAS discovery phase. The SNPs in each cluster are sorted by genomic position and the markers highlighted in bold were selected to represent their cluster in the technical validation stage (clusters 28, 30, 31 and 41 were not tested in the technical validation as primers for Sequenom genotyping could not be designed for any of the SNPs belonging to these clusters).
(DOCX)

S4 Table Top 100 SNPs in the PSP GWAS discovery phase according to the combined Z-test. The markers are sorted by increasing *P*-value and the top 49 SNPs highlighted in bold were selected for technical validation through this approach.
(DOCX)

S5 Table 101 SNPs from the GWAS discovery phase prioritized for technical validation using three approaches (|RASdiff|, cluster method and combined Z-test). The SNPs selected by the |RAS_diff_| method are listed first (48 SNPs), followed by those selected through the cluster method (total of 54 SNPs, 9 of which have already been selected by the |RAS_diff_| strategy) and finally the combined Z-test (total of 49 SNPs, 37 of which have already been selected by the previous two methods). For each SNP, the |RAS_diff_|, LD score/Cluster ID and *P*-value for a given SNP are only indicated if the SNP passed the selection threshold in the respective method.
(DOCX)

S6 Table Association results of the PSP GWAS technical validation phase. The 94 SNPs are ranked in increasing order of *P*-value and nominally significant associations are highlighted in bold.
(DOCX)
