# Supplementary figures and images for: Multicentric Genome-Wide Association Study for Primary Spontaneous Pneumothorax
Source: PLoS One. 2016 May 20;11(5):e0156103. doi: 10.1371/journal.pone.0156103 (PMC4874577; doi:10.1371/journal.pone.0156103)

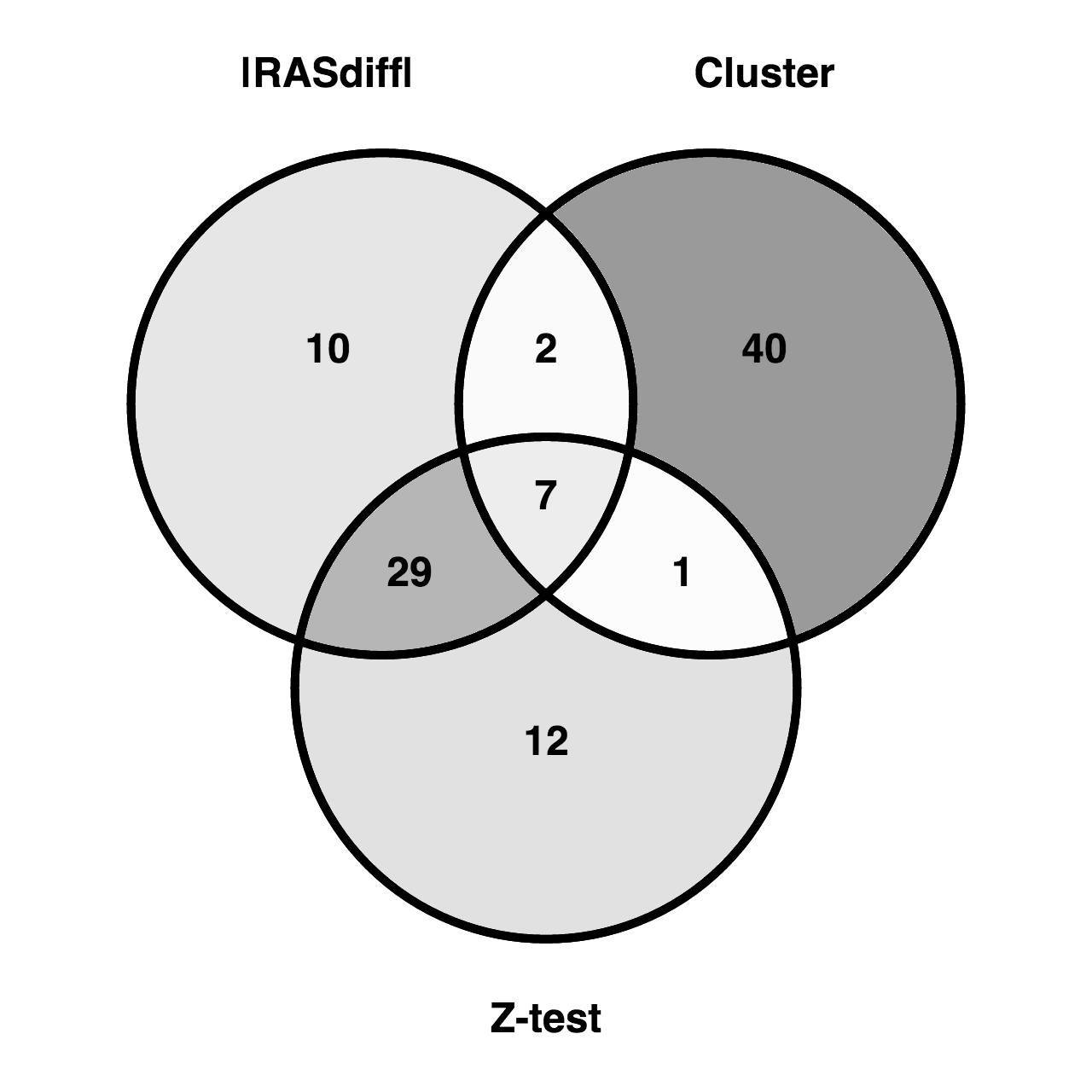

Supplement: S2 Fig — The numbers of SNPs chosen by each of the three approaches and overlapping among methods are indicated. (TIFF) [file pone.0156103.s002.tiff]
