# Supplementary material for: Multicentric Genome-Wide Association Study for Primary Spontaneous Pneumothorax
Source: PLoS One. 2016 May 20;11(5):e0156103. doi: 10.1371/journal.pone.0156103 (PMC4874577; doi:10.1371/journal.pone.0156103)
Supplement: S1 Table — (DOCX) [file pone.0156103.s003.docx]

**S1 Table. Primer sequences used to genotype the 101 SNPs studied in the technical validation phase.**

| **SNP** | **PCR primer 1** | **PCR primer 2** | **Extension primer** |
| --- | --- | --- | --- |
| **rs10508279** | ACGTTGGATGGAAGGTGAGGCAATATAAGG | ACGTTGGATGCTGAGCATGGTATCATAGAG | GGCAGATGAACGTCT |
| **rs2919427** | ACGTTGGATGCTTTCCGTCCCAGTAAAATG | ACGTTGGATGCTTCTCCGCGTTTGTGACTG | TGACAGCATCTCCTTC |
| **rs487013** | ACGTTGGATGCCGATAAGCAAATGCACACG | ACGTTGGATGGGGTGTAAATTTCTAGACG | TCAGAGCTGACAGAGG |
| **rs7374822** | ACGTTGGATGTCCACCTCTTTTCTTCTCCC | ACGTTGGATGGTCATTTCTTGGCAATGGTG | CATCTCCTAACCATCACA |
| **rs11629958** | ACGTTGGATGGAGTAGAAAGAGGACATTT | ACGTTGGATGCCTGCCTGGAACAGAAAATG | AGGACATTTCTAAAGGCA |
| **rs4434965** | ACGTTGGATGCAGAGCGCTTTCAGCAATTC | ACGTTGGATGCGTTAATTGTTAGTTTTCCTG | CTTTTGTCATATTGGACCC |
| **rs7741604** | ACGTTGGATGGTACCATGTTCTGGCTTCTG | ACGTTGGATGCTGCCTTCCATCTTCATTCC | ttgGCTGTGTTGAGACCAA |
| **rs1131535** | ACGTTGGATGCACTGTTCTGAAAGTGACTC | ACGTTGGATGACTGATATGAGGTAGAGTCC | acGACTCACTTATCCCAAGA |
| **rs2926721** | ACGTTGGATGGAGGTAAGATTCTCCACAGG | ACGTTGGATGGCTGAGTGAAGTGAACCTTG | gaAGGCCAGATGGATATTTA |
| **rs459020** | ACGTTGGATGTCCTAGAAGATACAAGAGGC | ACGTTGGATGTTTCAAAATTCTGTTGTGG | tCAAGTATTATCGAGCTTTCA |
| **rs7911954** | ACGTTGGATGTTCCCAAATGCTCCCTGAAG | ACGTTGGATGCTTTCCCTCACTACGCTTTG | CCATCAGGTGTAATAATCGTA |
| **rs11847260** | ACGTTGGATGTTGGGCAGCTGTAAAATGGG | ACGTTGGATGTAAAGCCCCAGATACCTTGC | gGTAAAATGGGAATAGTGCTT |
| **rs7767391** | ACGTTGGATGAAGCCCTGATCAGCTGTTAC | ACGTTGGATGGGCGAATGTGATAATTAGGG | CACCATCCAATTTGCAAATCTC |
| **rs9487033** | ACGTTGGATGATGTCTAGTCAAGAGGACCC | ACGTTGGATGTCTCCCTTTGCATTCTCCTC | CCACTTCAATCTAGATATCCTTA |
| **rs17671063** | ACGTTGGATGCTTCTAGGTTGCTACATGGC | ACGTTGGATGTTAAGAATGCTGGGAGGAGG | CCAACCCTTGACTTATTAAGAAT |
| **rs4602358** | ACGTTGGATGAATTGGCTGAAGGCAAAGGG | ACGTTGGATGCTAGACAATGGCTCTTATAG | tagtGACTAGAAAGAACTTTCCAG |
| **rs16855688** | ACGTTGGATGCTTTTGGTCCTGGAAAGGTG | ACGTTGGATGGGATAGTGTGTAATTCATGC | tggcAAGGTGAAGACCTTGATCCT |
| **rs6945688** | ACGTTGGATGGTCATTAACCCAGTATACCC | ACGTTGGATGAAGCGTGGATGAGGACTAGC | tttcTACCCTGTTGTTTTCCACTAA |
| **rs230833** | ACGTTGGATGACAAGTGGGGCCTTATATTG | ACGTTGGATGCCCCTTTCCTGCACTAATAC | ttccAGTGCTTTCATCATCAAAGAA |
| **rs6902892** | ACGTTGGATGGTCTGTTCACAGTTGATAGC | ACGTTGGATGCCCAGCTACTGACGTCATTT | aacACAATCTTCTCCACTTAATAAAT |
| **rs4883870** | ACGTTGGATGCTTCTGTCAACCTCCATGAG | ACGTTGGATGAAATAGGCCAGGCACAGAAG | gataATATTTTTTAGCCCCTATGTAA |
| **rs2101167** | ACGTTGGATGCAAGGTGATGAATGATGGTC | ACGTTGGATGTAACTGGGTCACATGCTGCG | aaactAATGATGGTCTATATTGGCAA |
| **rs7241671** | ACGTTGGATGTCAAGAAAACATGGAAATG | ACGTTGGATGATACAATGGGGCATAATAGG | aatAAAACATGGAAATGTTACATTTG |
| **rs13331343** | ACGTTGGATGGTAGTGATTCATTAATCTCC | ACGTTGGATGTGGGCACCTACCTTCTTTAG | cgttATTCATTAATCTCCTTAGCCTAT |
| **rs10504160** | ACGTTGGATGTTGAGAGCTTTCCAGAGCAG | ACGTTGGATGGCTGCATGCCTTATGTTGCC | gagcAGAACTAGTTGATTTTGATAAGA |
| **rs6466365** | ACGTTGGATGACACCAAGCAACAGAACTTC | ACGTTGGATGAAAAAGGCCACTCAGGGAAC | gccccAGCAACAGAACTTCAAAGACTTA |
| **rs2545886** | ACGTTGGATGAGTATCTACTTGCTCATTC | ACGTTGGATGGTTCACTGTACAATGTCTTCC | AATAATGTTTCATTATTATCAAACATAC |
| **rs13188604** | ACGTTGGATGAGGAAACTTCGTTGGTTGAG | ACGTTGGATGTCCTACTAATCCCTGCCATC | gCTGAAAAATATAAAGCAAAACTGTCTA |
| **rs10808167** | ACGTTGGATGTCTCTGAGGAAAAGAGTGGC | ACGTTGGATGGCATTGCTCATTGTTTCACTG | gggaCAAAATTAAAGAGGTAACTGTTAT |
| **rs10956847** | ACGTTGGATGCTGGGTTTGAAACCAGGTAG | ACGTTGGATGTCTCCCAACAATGGTAGCAG | TTCACCTGGCTCTCA |
| **rs17133680** | ACGTTGGATGTTACCTAGAGACAGAGGAGC | ACGTTGGATGACAACACAAGTTGCACTCAC | GAGGAGCCCTGTTTC |
| **rs2058487** | ACGTTGGATGCTGGCAGTAGAAAGCAGTTG | ACGTTGGATGAGAGATCAGTAAGGTGTAGC | tACAGCCACAGTGGGT |
| **rs17221652** | ACGTTGGATGCACAAGCCAGGGTCATAGG | ACGTTGGATGGGGAGAAGTGGATGGAAATC | GGTCATAGGACGATCAT |
| **rs1445324** | ACGTTGGATGGCAGGAGAATGAAGACCAAG | ACGTTGGATGTTGCTCCATTTGTCACCAAC | GACCAAGAAGGGGATAT |
| **rs4423896** | ACGTTGGATGCAATTCACCCAGGAACATGC | ACGTTGGATGAAACATGGGTGGGCCTTTAC | GAACATGCCTTTCTTCAT |
| **rs139167** | ACGTTGGATGTCTCTCCCTCCATCCTTAAC | ACGTTGGATGTCAGACCCTCTTTGCTGATG | CCGTGCCCACAATGAATAA |
| **rs752962** | ACGTTGGATGGTATATTCTAACAATCGCAC | ACGTTGGATGGGGAACCACGGTATGTTTAG | CGCACAAATAAAGATGCTA |
| **rs2413151** | ACGTTGGATGAGAAATGTACTCCCTCTCCC | ACGTTGGATGTTACTGGCCAAGGAAGACAC | TCCCCTTTCTAATTTTGACC |
| **rs922799** | ACGTTGGATGCAACCAAACATACACATGGG | ACGTTGGATGTGCTAACAGATGGTTTGGAC | aATGGGAAGCCATATGGTTT |
| **rs287916** | ACGTTGGATGAAATCCAGGACGTCCCAAAC | ACGTTGGATGCTTAATCCAGGCTTCCTCAG | cCGTCCCAAACATTTCTAAAC |
| **rs7463038** | ACGTTGGATGCATAAGGAGTACTCAGCTGG | ACGTTGGATGCGGCCACACAAGACGTTTTT | cccccACTCAGCTGGGTTCAC |
| **rs10222715** | ACGTTGGATGTGGGAGTGAGATTCCAAAGC | ACGTTGGATGGCAGTATTTTCTGAGTTAT | GCCTATTGCCAAAATGAGTCT |
| **rs1397056** | ACGTTGGATGGAATGTGATACACAGATGGG | ACGTTGGATGGTGACTGAGAGTTACTTCCC | CTACCTCACTAAGCTGTGTAAC |
| **rs4377469** | ACGTTGGATGATGCTATAAGAGCCATTGTC | ACGTTGGATGGTTCCAAAATTGCTCTAGGG | gAAGAGCCATTGTCTAGTATTG |
| **rs2122914** | ACGTTGGATGGTACTCAGCTTTTCAGTGGG | ACGTTGGATGTTTGGTGGTCAAGACATTCC | cccccGGACCGGATTTCAAATCT |
| **rs346501** | ACGTTGGATGGGGAAATCATTTTCAGTGACC | ACGTTGGATGACCTCTTCTGTCCTTTGCTG | ATTTTCAGTGACCTTAATTAGTA |
| **rs6983560** | ACGTTGGATGTAGGTGATAGAATTGTGGGC | ACGTTGGATGTGTTAGAGCACAGACAGCAC | gttgGGGCATACTTGTAGGAAGC |
| **rs8048056** | ACGTTGGATGGAAACAGCTCTGAGTCCTTC | ACGTTGGATGGCAACACAGAATCCTTTGGC | aTCTGAGTCCTTCTAGGATATTTT |
| **rs287903** | ACGTTGGATGCTTTGAGTTTAACTCAGCAC | ACGTTGGATGACGGCTGTCTTGGTATTTCC | ccccTTTCAATGTCTACAGTTGTTC |
| **rs1333199** | ACGTTGGATGCAGGCGTTATAGAATGGCAC | ACGTTGGATGAAGTCAAAGTCAGATTAGC | gTGGAAAACATTATATTGAAAGTTG |
| **rs411167** | ACGTTGGATGTGGGATTATACACCCATG | ACGTTGGATGAAAAGGGCTGGAAGAACTGG | cccgTGTTTATCCTCAAAGCAGTATT |
| **rs6531429** | ACGTTGGATGCATGTCAAAATCAGTAACTAC | ACGTTGGATGGCAGAGATATTTTCATTCC | CAAAATCAGTAACTACTTTAATGGAA |
| **rs3097903** | ACGTTGGATGCCATCCCATATGCATTGCTG | ACGTTGGATGAAAGATAACTCTTGAGAGC | ccccgACCTATGTACTATAACTCCATA |
| **rs10942788** | ACGTTGGATGCCTGTGTAGGTTTACCAAGC | ACGTTGGATGAGAAAGTGTGGGCTAGTATG | ggatGTTTACCAAGCTAATTATACTCT |
| **rs1526483** | ACGTTGGATGCCCTTGTCTGTAGAATAGAG | ACGTTGGATGTTGTGCCAAGACCTTTACAG | TCTGTAGAATAGAGATAATAATGGTGT |
| **rs4727161** | ACGTTGGATGTGCTTCATGGTGTAATTTGC | ACGTTGGATGCGTAGCAGACTATAAAGTTC | ggggTTCATGGTGTAATTTGCTGATAG |
| **rs7831961** | ACGTTGGATGGCTTCACTGGTGCTCAAAAC | ACGTTGGATGCGAGGTAAGTTCATTAGTAG | gggcTAGTTTCTTTTTCTGTTCTTAGAT |
| **rs4733649** | ACGTTGGATGATATCCATGTTCCAGCCAGC | ACGTTGGATGCTCTTTCAAATTAAGGAACC | gggtgCCAGCCAGCTGGCACAAATAACA |
| **rs11708202** | ACGTTGGATGTGAACTGTCCATGCTGACCG | ACGTTGGATGTGCTCACTAGTATGGAAGAG | GGGAAACAGGCTCAC |
| **rs3789950** | ACGTTGGATGAAGTTGGGCTTTCTGCTCAC | ACGTTGGATGCTCTGGCTTGGACAAGTCTA | CAAGGACAAAGTCCTCA |
| **rs10484048** | ACGTTGGATGGAGAACAGATAGTTGGCCAC | ACGTTGGATGGTGAGATGAGCTTGCAGGAG | cGTTGGCCACACAATTAT |
| **rs155681** | ACGTTGGATGAATACTTTTTGTGGCATGA | ACGTTGGATGGGGCACATGTACTGTGATTT | GATTGGGAACAAGCTAAG |
| **rs10491529** | ACGTTGGATGAGTGACAGCATCCTGTATTG | ACGTTGGATGAGATACTGTTTGCAAATCGG | AAGATTTCACACGAGTCTT |
| **rs4397338** | ACGTTGGATGGGGAAACATATTTGCAAGC | ACGTTGGATGGACCACTTTTTAGGCTATTG | ggtgTTTGCAAGCCAGGTA |
| **rs1353318** | ACGTTGGATGCACATGTCCATGGCATGGCT | ACGTTGGATGGAACTCTTGCCTCATGTCTG | TCCATGGCATGGCTATATAA |
| **rs9656970** | ACGTTGGATGACCTAAACCTTTGGTCGCTG | ACGTTGGATGGAGTTCCTGGAGGTCTGTTT | cccCCGTTCCCAGTTGTCGTT |
| **rs10903913** | ACGTTGGATGCCCCAGATGTCTCTGCTATA | ACGTTGGATGGGGCCAAAAAATATCACTCC | GTCTCTGCTATAAAAGTTCTG |
| **rs612389** | ACGTTGGATGCAAGGGAAGGATTCATAGAC | ACGTTGGATGTCCACATGCTCTTGCTAACG | ggggTCTGCATCTCAATAGGG |
| **rs10825483** | ACGTTGGATGCAGAAGTAAATGGCCTAGTC | ACGTTGGATGTGCAGAGTATAAATGGACAG | gGACAGCCAAATTTATATCTCA |
| **rs2164512** | ACGTTGGATGGGGAATGGATGAGCCTTATG | ACGTTGGATGGTAGACTTCCTGTTGCAATC | agGGATGAGCCTTATGTAAAGA |
| **rs7682400** | ACGTTGGATGTGTCCCCTCTCAAATGTCAC | ACGTTGGATGAGGGTGTGAGTGTTCTCTTG | cATGTCACACTGATGTGATTGTA |
| **rs2971955** | ACGTTGGATGCCCACCAATTACACAGCTAC | ACGTTGGATGTCTGGAAGAAGAAGCTGACC | aaAGCTACTCATGGTGATGATCT |
| **rs4602638** | ACGTTGGATGGCTGTGTGGCTGCAAGAAAG | ACGTTGGATGAACCAGAAGGGATGTGAGAG | cccacCTCTGGAGTCCAGTTTCTT |
| **rs12792701** | ACGTTGGATGTTTGATATCAGGTCTGGAGC | ACGTTGGATGCCAAGGAAAATTTACGTTGC | ggaatGTCTGGAGCTATATGTACT |
| **rs4457905** | ACGTTGGATGCATCATTACTGAAAATTGGC | ACGTTGGATGGCAAGGGTCCCAAGCATTAC | cccgACTGAAAATTGGCAGTTCTTC |
| **rs6881724** | ACGTTGGATGGCAAGCCTGAAGAAAAAGAG | ACGTTGGATGAATAGGGATGCCCTTGTCTG | agcAAAAGAGGTTCTATTATCTTCC |
| **rs8083684** | ACGTTGGATGACATAGAGGAAACATGTGAC | ACGTTGGATGCCCCATGTTATTACAAACTTC | gaggGAGGAAACATGTGACCAAATT |
| **rs1525833** | ACGTTGGATGCATGCTTTAATGACTATTG | ACGTTGGATGGGTCTATACATGAGTAAAG | gggGCTTTAATGACTATTGTCATCAA |
| **rs10088760** | ACGTTGGATGTATACCTGTACTTCATTTG | ACGTTGGATGCTAGGAAATACCAGCAGGAG | CCTGTACTTCATTTGTTAATTCCAATA |
| **rs17491067** | ACGTTGGATGGAATACTATGTAGTAGTTC | ACGTTGGATGATTGCCACAGAAACTAGAAC | tgagACTATGTAGTAGTTCTTCCACTC |
| **rs2041301** | ACGTTGGATGAGAAGCCTGACAAAGCAGAC | ACGTTGGATGATTATGCAGACTGCCTTGCC | ctaaAAAGCAGACTTTAGAGAGAAAAA |
| **rs9547912** | ACGTTGGATGATAGAAAGCAGGAGCATCCA | ACGTTGGATGATGCTGTACTGTGGCAAAGG | agagcGAAAGCAGGAGCATCCATATAAC |
| **rs6110533** | ACGTTGGATGAGCGGAGAACACAAGAAGAC | ACGTTGGATGATCATCTCTGCCAGGAAACC | AGAAGACCATGGAGAAG |
| **rs236715** | ACGTTGGATGGCACTCTAATGTCTCAATGG | ACGTTGGATGGAAGCTTTGGAAGGTCACTG | GCAACAAGAATACCTTGC |
| **rs488940** | ACGTTGGATGAGCGGTGGTGTTTCTTTGAC | ACGTTGGATGGGGAGAGAGTTTATGGTGTC | CCTTGAAAGTCACGTAGAA |
| **rs4922683** | ACGTTGGATGTTGTGTTGCTGTTTTGACTG | ACGTTGGATGGTCACGTATTATATAGATCC | tTGTTTTGACTGTCTCCTTA |
| **rs436563** | ACGTTGGATGGGTGAAATGTCTAGAAACCC | ACGTTGGATGCCCTTAAGTCTTTCCTCCAG | TCTAGAAACCCCAAAATATGA |
| **rs1962137** | ACGTTGGATGCGGAGGTATTTCCGTTTGTG | ACGTTGGATGTCAGCAGGATGGAGGAAAAG | GTCTAATTGTTGCTCTTATGG |
| **rs10966315** | ACGTTGGATGTATCCTGTTCTGCATGTGAC | ACGTTGGATGCATTCAAAGATGCATTGGTC | gGACAGGATATTTAGGTTCTTT |
| **rs12583307** | ACGTTGGATGATAGACCGATGTACCAAGGC | ACGTTGGATGGGGACATGTACATTGTATTCC | ggggCCAAGGCAGTAAAACATC |
| **rs723436** | ACGTTGGATGGCCCAGGTTGTATTTATGAG | ACGTTGGATGTCGTCAACTTCCTGTCCATC | TGCATTTATGTACCCATTTATAT |
| **rs9423526** | ACGTTGGATGCCCTTCAGTGAGTCTTAGTC | ACGTTGGATGCAGTGAGAAGTAACTGCTTG | ttctTGAGTCTTAGTCAATTTGC |
| **rs10820588** | ACGTTGGATGACGTTTACCCAAGGCATGAG | ACGTTGGATGGGCACAAAGCAGATGTCACC | ggTGAATTTGGAGAATCAATGAA |
| **rs10242076** | ACGTTGGATGGAGAAAATTGAAGCTTAAATC | ACGTTGGATGCGTTATCTCAGTATGCTGCC | AAATTGAAGCTTAAATCTATTAGG |
| **rs2962615** | ACGTTGGATGTCTCTCAACCACTGTCTGCC | ACGTTGGATGTAGAGCCCCTGAAAGTTGTG | ctCAACTGGAAAATTATCTCTAAAA |
| **rs516081** | ACGTTGGATGGTTCAGTAAATGTTTAAGGAG | ACGTTGGATGGGTACTTGGTAGTCTATTTC | cAATGAATAAATACATTGCCAAAAGT |
| **rs9522832** | ACGTTGGATGCCACTTCCAGATCCTAACAG | ACGTTGGATGAATACATCTTCAACAGTCC | atgAAAAGAACAGACACTGAAAAGTC |
| **rs1495980** | ACGTTGGATGCAGGTGGAAGCTAAGCTATG | ACGTTGGATGCCCACTCTCTCTTTTGAGTC | gagagAAGCTATGGTTACATAGGAAC |
| **rs12027334** | ACGTTGGATGTACGGCAAATGATCAAGAGG | ACGTTGGATGTGGAGAGAGAGCACTTCAAG | ccccCAGACTAAAATGTGATTCTCAAC |
| **rs8055491** | ACGTTGGATGCCCAGACACAATAGTGGAGC | ACGTTGGATGTCCCACATGTTATCTTCCTG | TGTTTTTAAGAAATATTTTATTACCCAA |
| **rs12666340** | ACGTTGGATGCCCCATGGTTAGCATGTTTC | ACGTTGGATGCAGTACATGGTCATAGTCCC | ggccTGGTTAGCATGTTTCATTCAAAAT |
