# Supplementary material for: Multicentric Genome-Wide Association Study for Primary Spontaneous Pneumothorax
Source: PLoS One. 2016 May 20;11(5):e0156103. doi: 10.1371/journal.pone.0156103 (PMC4874577; doi:10.1371/journal.pone.0156103)
Supplement: S2 Table — The SNPs are sorted by decreasing |RASdiff|, then by chromosomal position, and the top 48 markers highlighted in bold were selected for the technical validation stage. (DOCX) [file pone.0156103.s004.docx]

**S2 Table. SNPs with |RASdiff|>12% in the PSP GWAS discovery phase.** The SNPs are sorted by decreasing |RAS_diff_|, then by chromosomal position, and the top 48 markers highlighted in bold were selected for the technical validation stage.

| Affymetrix SNP ID | SNP | Chr. | Position (bp) | Gene | MAF | \|RAS_diff_\| |
| --- | --- | --- | --- | --- | --- | --- |
| SNP_A-8284151 | **rs10504160** | 8 | 55013096 | *LYPLA1* | 0.000 | 0.191 |
| SNP_A-1906024 | **rs2101167** | 3 | 156691408 | *LEKR1* | 0.067 | 0.167 |
| SNP_A-4303960 | **rs7741604** | 6 | 20731524 | *CDKAL1* | 0.083 | 0.162 |
| SNP_A-4207170 | **rs2919427** | 16 | 30669091 | *-* | 0.000 | 0.162 |
| SNP_A-1988278 | **rs287903** | 6 | 157164550 | *ARID1B* | 0.192 | 0.159 |
| SNP_A-1953716 | **rs4922683** | 11 | 24535089 | *LUZP2* | 0.133 | 0.158 |
| SNP_A-2129421 | **rs4423896** | 4 | 78340170 | *-* | 0.367 | 0.157 |
| SNP_A-2172641 | **rs6902892** | 6 | 109627601 | *CCDC162* | 0.475 | 0.156 |
| SNP_A-8370747 | **rs17671063** | 7 | 68381395 | *-* | 0.300 | 0.154 |
| SNP_A-8670099 | **rs6466365** | 7 | 110577992 | *IMMP2L* | 0.267 | 0.153 |
| SNP_A-8643425 | **rs1131535** | 3 | 172224075 | *TNFSF10* | 0.392 | 0.150 |
| SNP_A-1845593 | **rs4602358** | 3 | 42303037 | *CCK* | 0.450 | 0.149 |
| SNP_A-1926346 | **rs2545886** | 16 | 21029197 | *DNAH3* | 0.100 | 0.148 |
| SNP_A-1923579 | **rs7767391** | 6 | 20725240 | *CDKAL1* | 0.142 | 0.147 |
| SNP_A-8393894 | **rs459020** | 6 | 95052842 | *-* | 0.175 | 0.146 |
| SNP_A-1862608 | **rs7831961** | 8 | 135507060 | *ZFAT* | 0.242 | 0.146 |
| SNP_A-8367558 | **rs6531429** | 4 | 36275383 | *-* | 0.292 | 0.145 |
| SNP_A-8385981 | **rs752962** | 10 | 14177928 | *FRMD4A* | 0.292 | 0.144 |
| SNP_A-1804934 | **rs17221652** | 10 | 1416472 | *ADARB2* | 0.375 | 0.143 |
| SNP_A-1852386 | **rs10222715** | 4 | 57614160 | *-* | 0.333 | 0.142 |
| SNP_A-2047275 | **rs10942788** | 5 | 75957603 | *IQGAP2* | 0.483 | 0.141 |
| SNP_A-8475662 | **rs1333199** | 10 | 2185200 | *-* | 0.192 | 0.141 |
| SNP_A-8604971 | **rs11629958** | 15 | 84147713 | *SH3GL3* | 0.375 | 0.141 |
| SNP_A-4254240 | **rs139167** | 22 | 44598692 | *PARVG* | 0.208 | 0.140 |
| SNP_A-2151292 | **rs7682400** | 4 | 182292227 | *-* | 0.233 | 0.138 |
| SNP_A-2117404 | **rs7911954** | 10 | 2160519 | *-* | 0.208 | 0.138 |
| SNP_A-1874570 | **rs7374822** | 3 | 134820184 | *EPHB1* | 0.400 | 0.137 |
| SNP_A-1814946 | **rs10903913** | 10 | 3042256 | *-* | 0.083 | 0.137 |
| SNP_A-1992858 | **rs10508279** | 10 | 4300734 | *-* | 0.083 | 0.137 |
| SNP_A-4269357 | **rs1445324** | 11 | 27034608 | *-* | 0.350 | 0.137 |
| SNP_A-2066116 | **rs12792701** | 11 | 28988343 | *-* | 0.142 | 0.136 |
| SNP_A-8591019 | **rs7241671** | 18 | 72764169 | *ZNF407* | 0.142 | 0.136 |
| SNP_A-2170991 | **rs3097903** | 4 | 124766331 | *LOC285419* | 0.342 | 0.135 |
| SNP_A-4275369 | **rs9487033** | 6 | 109605388 | *-* | 0.417 | 0.135 |
| SNP_A-8298577 | **rs4733649** | 8 | 129798114 | *-* | 0.325 | 0.135 |
| SNP_A-2112567 | **rs723436** | 13 | 22331539 | *-* | 0.367 | 0.135 |
| SNP_A-2037621 | **rs236715** | 20 | 57697095 | *-* | 0.125 | 0.135 |
| SNP_A-1936662 | **rs4602638** | 5 | 31122961 | *-* | 0.425 | 0.134 |
| SNP_A-4207233 | **rs10088760** | 8 | 112341321 | *-* | 0.292 | 0.134 |
| SNP_A-8685522 | **rs6881724** | 5 | 97846394 | *-* | 0.192 | 0.133 |
| SNP_A-8530724 | **rs1962137** | 11 | 89990909 | *-* | 0.217 | 0.133 |
| SNP_A-2036636 | **rs4457905** | 14 | 30233422 | *PRKD1* | 0.208 | 0.133 |
| SNP_A-8463662 | **rs1526483** | 7 | 83506643 | *-* | 0.158 | 0.132 |
| SNP_A-4293603 | **rs2971955** | 7 | 133665939 | *EXOC4* | 0.317 | 0.132 |
| SNP_A-8562522 | **rs10956847** | 8 | 93498901 | *-* | 0.267 | 0.132 |
| SNP_A-1929490 | **rs6983560** | 8 | 135584553 | *ZFAT* | 0.208 | 0.132 |
| SNP_A-8311750 | **rs2058487** | 9 | 122945873 | *-* | 0.242 | 0.132 |
| SNP_A-1882930 | **rs488940** | 11 | 55261905 | *-* | 0.367 | 0.132 |
| SNP_A-2107533 | rs16906858 | 12 | 30983129 | *-* | 0.200 | 0.132 |
| SNP_A-8427831 | rs155123 | 2 | 182312272 | *-* | 0.442 | 0.131 |
| SNP_A-8395729 | rs6946494 | 7 | 135488621 | *-* | 0.175 | 0.131 |
| SNP_A-8518753 | rs4883870 | 13 | 70940325 | *-* | 0.483 | 0.131 |
| SNP_A-4206508 | rs12870439 | 13 | 70947913 | *-* | 0.283 | 0.131 |
| SNP_A-4289018 | rs7774390 | 6 | 109602461 | *-* | 0.475 | 0.130 |
| SNP_A-1876776 | rs287916 | 6 | 157159008 | *ARID1B* | 0.192 | 0.130 |
| SNP_A-8292508 | rs4978396 | 9 | 112269480 | *-* | 0.342 | 0.130 |
| SNP_A-8285645 | rs10491529 | 9 | 122932725 | *-* | 0.242 | 0.130 |
| SNP_A-8665296 | rs428751 | 5 | 101465106 | *-* | 0.458 | 0.129 |
| SNP_A-8318599 | rs2228211 | 6 | 12122773 | *HIVEP1* | 0.233 | 0.129 |
| SNP_A-4273197 | rs7018093 | 8 | 129891232 | *-* | 0.317 | 0.129 |
| SNP_A-2271057 | rs4354342 | 8 | 135273013 | *-* | 0.283 | 0.129 |
| SNP_A-4225651 | rs6064503 | 20 | 37026379 | *-* | 0.117 | 0.129 |
| SNP_A-4298254 | rs3109359 | 4 | 112374964 | *-* | 0.117 | 0.128 |
| SNP_A-8557191 | rs12666340 | 7 | 120716152 | *C7orf58* | 0.467 | 0.128 |
| SNP_A-8627636 | rs13277153 | 8 | 129708542 | *-* | 0.258 | 0.128 |
| SNP_A-8580078 | rs6470649 | 8 | 129726384 | *-* | 0.258 | 0.128 |
| SNP_A-2054267 | rs733254 | 8 | 135638632 | *ZFAT* | 0.283 | 0.128 |
| SNP_A-8640336 | rs1547299 | 13 | 71001032 | *-* | 0.317 | 0.128 |
| SNP_A-2026110 | rs2962615 | 5 | 2689899 | *-* | 0.492 | 0.127 |
| SNP_A-8423678 | rs1525833 | 7 | 46069653 | *-* | 0.433 | 0.127 |
| SNP_A-8322649 | rs4718600 | 7 | 67057015 | *-* | 0.192 | 0.127 |
| SNP_A-8283740 | rs1353318 | 8 | 15338089 | *-* | 0.158 | 0.127 |
| SNP_A-2144398 | rs10966315 | 9 | 24467996 | *-* | 0.092 | 0.127 |
| SNP_A-8402116 | rs487013 | 9 | 112831877 | *AKAP2* | 0.450 | 0.127 |
| SNP_A-8509416 | rs612389 | 11 | 84724185 | *DLG2* | 0.142 | 0.127 |
| SNP_A-8467764 | rs29035 | 18 | 9996143 | *-* | 0.117 | 0.127 |
| SNP_A-1807749 | rs2987838 | 10 | 25778873 | *GPR158* | 0.467 | 0.126 |
| SNP_A-1826262 | rs2144862 | 10 | 52260398 | *SGMS1* | 0.433 | 0.126 |
| SNP_A-1836573 | rs13077330 | 3 | 28761836 | *-* | 0.483 | 0.125 |
| SNP_A-2155416 | rs16855688 | 3 | 170772678 | *-* | 0.092 | 0.125 |
| SNP_A-2278249 | rs2164512 | 16 | 64082446 | *-* | 0.225 | 0.125 |
| SNP_A-2125156 | rs133561 | 22 | 48639063 | *-* | 0.192 | 0.125 |
| SNP_A-1795987 | rs1290558 | 1 | 118065841 | *MAN1A2* | 0.292 | 0.124 |
| SNP_A-2287650 | rs10865069 | 2 | 106391913 | *NCK2* | 0.200 | 0.124 |
| SNP_A-2087314 | rs2317966 | 6 | 1550886 | *-* | 0.375 | 0.124 |
| SNP_A-2035616 | rs7463038 | 8 | 38587043 | *TACC1* | 0.100 | 0.124 |
| SNP_A-8283802 | rs4961131 | 8 | 90404592 | *-* | 0.450 | 0.124 |
| SNP_A-8609539 | rs10088689 | 8 | 112341055 | *-* | 0.292 | 0.124 |
| SNP_A-1968607 | rs10485620 | 20 | 50565168 | *-* | 0.167 | 0.124 |
| SNP_A-8360659 | rs4377469 | 3 | 42303074 | *CCK* | 0.083 | 0.123 |
| SNP_A-1959731 | rs6533232 | 4 | 106909442 | *NPNT* | 0.367 | 0.123 |
| SNP_A-4249625 | rs9348123 | 6 | 166771814 | *-* | 0.300 | 0.123 |
| SNP_A-8435666 | rs17417090 | 7 | 78047073 | *MAGI2* | 0.250 | 0.123 |
| SNP_A-8535257 | rs8055491 | 16 | 52824235 | *-* | 0.433 | 0.123 |
| SNP_A-1960639 | rs6663840 | 1 | 3743319 | *KIAA0562* | 0.325 | 0.122 |
| SNP_A-2258793 | rs17562236 | 1 | 75374025 | *-* | 0.283 | 0.122 |
| SNP_A-2273752 | rs10874963 | 1 | 96343561 | *-* | 0.383 | 0.122 |
| SNP_A-8346164 | rs9943186 | 1 | 210684455 | *HHAT* | 0.417 | 0.122 |
| SNP_A-4210587 | rs3775640 | 4 | 143047463 | *INPP4B* | 0.125 | 0.122 |
| SNP_A-8498929 | rs13182117 | 5 | 42611848 | *GHR* | 0.158 | 0.122 |
| SNP_A-1950562 | rs230833 | 5 | 56915585 | *-* | 0.225 | 0.122 |
| SNP_A-2159777 | rs13361218 | 5 | 58470077 | *PDE4D* | 0.258 | 0.122 |
| SNP_A-8328738 | rs13156451 | 5 | 109306552 | *-* | 0.358 | 0.122 |
| SNP_A-1852457 | rs153580 | 5 | 115683307 | *-* | 0.217 | 0.122 |
| SNP_A-8539193 | rs4705965 | 5 | 132055127 | *KIF3A* | 0.292 | 0.122 |
| SNP_A-2199424 | rs9369299 | 6 | 41619689 | *MDFI* | 0.333 | 0.122 |
| SNP_A-2093234 | rs1111866 | 6 | 109610846 | *-* | 0.417 | 0.122 |
| SNP_A-2292779 | rs364583 | 8 | 37159346 | *-* | 0.208 | 0.122 |
| SNP_A-2005775 | rs4605265 | 18 | 67255632 | *DOK6* | 0.100 | 0.122 |
| SNP_A-8390264 | rs870843 | 3 | 39435865 | *SLC25A38* | 0.300 | 0.121 |
| SNP_A-1838900 | rs1490208 | 6 | 77169787 | *-* | 0.283 | 0.121 |
| SNP_A-2097982 | rs7772602 | 6 | 109608661 | *-* | 0.417 | 0.121 |
| SNP_A-2238659 | rs2926610 | 8 | 76305778 | *-* | 0.333 | 0.121 |
| SNP_A-8417175 | rs17702348 | 10 | 115620229 | *NHLRC2* | 0.492 | 0.121 |
| SNP_A-4195069 | rs10896511 | 11 | 56462593 | *OR8U8* | 0.250 | 0.121 |
| SNP_A-8497541 | rs8008377 | 14 | 60082404 | *RTN1* | 0.200 | 0.121 |
| SNP_A-8341307 | rs2835954 | 21 | 39146427 | *KCNJ6* | 0.325 | 0.121 |
| SNP_A-1929844 | rs1250125 | 4 | 1192243 | *LOC100130872* | 0.300 | 0.120 |
| SNP_A-1809226 | rs346501 | 4 | 86854664 | *ARHGAP24* | 0.500 | 0.120 |
| SNP_A-4286518 | rs2850611 | 4 | 101904205 | *-* | 0.233 | 0.120 |
| SNP_A-4274295 | rs4691276 | 4 | 167359900 | *-* | 0.425 | 0.120 |
| SNP_A-8368745 | rs6887429 | 5 | 117212923 | *-* | 0.425 | 0.120 |
| SNP_A-4211837 | rs10498978 | 6 | 92895021 | *-* | 0.158 | 0.120 |
| SNP_A-8614939 | rs4727161 | 7 | 87988026 | *-* | 0.325 | 0.120 |
| SNP_A-4212876 | rs17422356 | 8 | 26558113 | *-* | 0.125 | 0.120 |
| SNP_A-4276804 | rs541661 | 8 | 27551229 | *-* | 0.442 | 0.120 |
| SNP_A-2040316 | rs9886652 | 8 | 106570222 | *ZFPM2* | 0.408 | 0.120 |
| SNP_A-2172772 | rs1929420 | 9 | 112989929 | *-* | 0.467 | 0.120 |
| SNP_A-2156877 | rs498102 | 9 | 113593899 | *-* | 0.192 | 0.120 |
| SNP_A-2198642 | rs931028 | 10 | 71373767 | *-* | 0.258 | 0.120 |
| SNP_A-8366741 | rs4938931 | 11 | 60026613 | *-* | 0.458 | 0.120 |
| SNP_A-2191281 | rs4544128 | 13 | 40868072 | *-* | 0.458 | 0.120 |
| SNP_A-8566828 | rs152712 | 16 | 49360358 | *-* | 0.458 | 0.120 |
| SNP_A-2081076 | rs4356573 | 18 | 61850900 | *LOC284294* | 0.383 | 0.120 |
| SNP_A-8504118 | rs848751 | 22 | 49725880 | *-* | 0.342 | 0.120 |

Abbreviations - Chr.: Chromosome; bp: Base pair position (Affymetrix GenomeWideSNP_6 Annotations, release 32); MAF: Minor allele frequency in the CEU HapMap samples (Affymetrix GenomeWideSNP_6 Annotations, release 32); |RAS_diff_|: Absolute value of the relative allele score difference between cases and controls.
