# Supplementary material for: Multicentric Genome-Wide Association Study for Primary Spontaneous Pneumothorax
Source: PLoS One. 2016 May 20;11(5):e0156103. doi: 10.1371/journal.pone.0156103 (PMC4874577; doi:10.1371/journal.pone.0156103)
Supplement: S3 Table — The SNPs in each cluster are sorted by genomic position and the markers highlighted in bold were selected to represent their cluster in the technical validation stage (clusters 28, 30, 31 and 41 were not tested in the technical validation as primers for Sequenom genotyping could not be designed for any of the SNPs belonging to these clusters). (DOCX) [file pone.0156103.s005.docx]

**S3 Table. SNP composition of the 54 genic and intergenic clusters with LD score ≥ 5 in the PSP GWAS discovery phase.** The SNPs in each cluster are sorted by genomic position and the markers highlighted in bold were selected to represent their cluster in the technical validation stage (clusters 28, 30, 31 and 41 were not tested in the technical validation as primers for Sequenom genotyping could not be designed for any of the SNPs belonging to these clusters).

| **Cluster ID** | **LD score** | **Gene** | **Chr.** | **SNP composition** | **Position (bp)** | **\|RAS_diff_\|** | **MAF** |
| --- | --- | --- | --- | --- | --- | --- | --- |
| 1 | 21 | *CSMD1* | 8 | rs2551040 | 3209943 | 0.091 | 0.283 |
|  |  |  |  | rs4487803 | 3219355 | 0.085 | 0.300 |
|  |  |  |  | rs6996400 | 3421997 | 0.085 | 0.317 |
|  |  |  |  | rs13258825 | 3423073 | 0.088 | 0.375 |
|  |  |  |  | rs6558803 | 3435939 | 0.090 | 0.475 |
|  |  |  |  | rs2291222 | 3474709 | 0.080 | 0.450 |
|  |  |  |  | rs4601341 | 3481560 | 0.085 | 0.433 |
|  |  |  |  | **rs922799** | 3481755 | 0.106 | 0.433 |
|  |  |  |  | rs6996513 | 3627151 | 0.084 | 0.333 |
|  |  |  |  | rs812941 | 4131490 | 0.081 | 0.292 |
|  |  |  |  | rs9314522 | 4165205 | 0.090 | 0.175 |
|  |  |  |  | rs6984591 | 4166781 | 0.082 | 0.250 |
|  |  |  |  | rs17336844 | 4316054 | 0.093 | 0.242 |
|  |  |  |  | rs2043804 | 4350040 | 0.104 | 0.150 |
|  |  |  |  | rs4875364 | 4444592 | 0.097 | 0.175 |
|  |  |  |  | rs2724985 | 4448387 | 0.080 | 0.450 |
|  |  |  |  | rs2725023 | 4481561 | 0.084 | 0.367 |
|  |  |  |  | rs17070881 | 4484362 | 0.087 | 0.092 |
|  |  |  |  | rs7839315 | 4960351 | 0.083 | 0.475 |
|  |  |  |  | rs6980727 | 4974195 | 0.086 | 0.500 |
|  |  |  |  | rs17351125 | 4992706 | 0.082 | 0.292 |
|  |  |  |  | rs4875441 | 5000769 | 0.088 | 0.433 |
| 2 | 11 | *ZFAT* | 8 | rs4909605 | 135498161 | 0.090 | 0.333 |
|  |  |  |  | rs7812708 | 135500832 | 0.100 | 0.183 |
|  |  |  |  | **rs7831961** | 135507060 | 0.146 | 0.242 |
|  |  |  |  | rs7822348 | 135542483 | 0.090 | 0.225 |
|  |  |  |  | rs6988000 | 135559534 | 0.112 | 0.183 |
|  |  |  |  | rs6578237 | 135569358 | 0.085 | 0.317 |
|  |  |  |  | rs7846013 | 135569967 | 0.085 | 0.305 |
|  |  |  |  | rs11166592 | 135577008 | 0.089 | 0.383 |
|  |  |  |  | rs6998749 | 135584316 | 0.099 | 0.208 |
|  |  |  |  | rs6983560 | 135584553 | 0.132 | 0.208 |
|  |  |  |  | rs11998030 | 135585325 | 0.087 | 0.208 |
|  |  |  |  | rs11780632 | 135606615 | 0.108 | 0.183 |
|  |  |  |  | rs11992096 | 135621369 | 0.111 | 0.342 |
|  |  |  |  | rs16905197 | 135625092 | 0.087 | 0.283 |
|  |  |  |  | rs733254 | 135638632 | 0.128 | 0.283 |
| 3 | 11 | *RBFOX1* | 16 | **rs8048056** | 6110691 | 0.115 | 0.308 |
|  |  |  |  | rs1424118 | 6111073 | 0.102 | 0.200 |
|  |  |  |  | rs4360938 | 6116418 | 0.088 | 0.117 |
|  |  |  |  | rs1463243 | 6181344 | 0.100 | 0.283 |
|  |  |  |  | rs9938831 | 6237017 | 0.113 | 0.092 |
|  |  |  |  | rs809684 | 6382434 | 0.082 | 0.242 |
|  |  |  |  | rs12930520 | 6697562 | 0.102 | 0.217 |
|  |  |  |  | rs11860754 | 6792419 | 0.089 | 0.108 |
|  |  |  |  | rs9924021 | 6813671 | 0.087 | 0.117 |
|  |  |  |  | rs8047052 | 7320496 | 0.082 | 0.250 |
|  |  |  |  | rs8051518 | 7320515 | 0.092 | 0.242 |
|  |  |  |  | rs10500355 | 7459347 | 0.085 | 0.317 |
| 4 | 10 | *CDKAL1* | 6 | rs4291090 | 20570039 | 0.094 | 0.300 |
|  |  |  |  | rs9368204 | 20580893 | 0.091 | 0.300 |
|  |  |  |  | rs9465837 | 20624179 | 0.105 | 0.125 |
|  |  |  |  | rs9465851 | 20640316 | 0.080 | 0.250 |
|  |  |  |  | rs6456368 | 20659806 | 0.099 | 0.308 |
|  |  |  |  | rs7754840 | 20661250 | 0.090 | 0.308 |
|  |  |  |  | rs7756992 | 20679709 | 0.106 | 0.250 |
|  |  |  |  | rs1040558 | 20713706 | 0.086 | 0.150 |
|  |  |  |  | rs9295478 | 20716253 | 0.096 | 0.200 |
|  |  |  |  | rs10946403 | 20717404 | 0.113 | 0.150 |
|  |  |  |  | rs2328549 | 20718240 | 0.084 | 0.092 |
|  |  |  |  | **rs7767391** | 20725240 | 0.147 | 0.142 |
|  |  |  |  | rs7741604 | 20731524 | 0.162 | 0.083 |
| 5 | 10 | *MAGI2* | 7 | rs17417090 | 78047073 | 0.123 | 0.250 |
|  |  |  |  | rs10485902 | 78121406 | 0.087 | 0.102 |
|  |  |  |  | rs1030014 | 78139428 | 0.084 | 0.400 |
|  |  |  |  | rs2190664 | 78322126 | 0.081 | 0.450 |
|  |  |  |  | rs4730461 | 78324858 | 0.094 | 0.442 |
|  |  |  |  | rs1118936 | 78366438 | 0.087 | 0.408 |
|  |  |  |  | **rs10808167** | 78366575 | 0.108 | 0.408 |
|  |  |  |  | rs1360302 | 78375066 | 0.088 | 0.483 |
|  |  |  |  | rs1888239 | 78390538 | 0.086 | 0.483 |
|  |  |  |  | rs4727761 | 78461880 | 0.081 | 0.108 |
|  |  |  |  | rs17455327 | 78469638 | 0.094 | 0.100 |
| 6 | 10 | *CDH13* | 16 | rs16957935 | 82675866 | 0.091 | 0.358 |
|  |  |  |  | rs7184633 | 82822013 | 0.080 | 0.367 |
|  |  |  |  | rs1559439 | 83310923 | 0.098 | 0.250 |
|  |  |  |  | rs2042434 | 83310978 | 0.091 | 0.117 |
|  |  |  |  | rs1862831 | 83323634 | 0.099 | 0.292 |
|  |  |  |  | rs1424189 | 83330610 | 0.090 | 0.350 |
|  |  |  |  | rs13337699 | 83375743 | 0.082 | 0.392 |
|  |  |  |  | rs7199636 | 83429902 | 0.092 | 0.458 |
|  |  |  |  | **rs13331343** | 83441188 | 0.115 | 0.442 |
|  |  |  |  | rs13336833 | 83515750 | 0.101 | 0.333 |
| 7 | 9 | *PTPRD* | 9 | rs7045605 | 8262469 | 0.108 | 0.186 |
|  |  |  |  | rs10815845 | 8387513 | 0.084 | 0.250 |
|  |  |  |  | rs12235754 | 8402449 | 0.088 | 0.375 |
|  |  |  |  | rs447925 | 10089748 | 0.096 | 0.242 |
|  |  |  |  | **rs436563** | 10100851 | 0.102 | 0.300 |
|  |  |  |  | rs439467 | 10102903 | 0.100 | 0.358 |
|  |  |  |  | rs294868 | 10124920 | 0.083 | 0.250 |
|  |  |  |  | rs294852 | 10185596 | 0.094 | 0.308 |
|  |  |  |  | rs12345788 | 10348933 | 0.081 | 0.250 |
| 8 | 9 | *FRMD4A* | 10 | rs6602676 | 13770065 | 0.088 | 0.433 |
|  |  |  |  | rs17549892 | 13962880 | 0.095 | 0.183 |
|  |  |  |  | rs2797860 | 13967167 | 0.099 | 0.392 |
|  |  |  |  | rs10906541 | 13971726 | 0.118 | 0.425 |
|  |  |  |  | rs2797890 | 13974185 | 0.094 | 0.250 |
|  |  |  |  | rs1026580 | 13981162 | 0.095 | 0.458 |
|  |  |  |  | rs11258878 | 14166989 | 0.090 | 0.333 |
|  |  |  |  | rs7914952 | 14169162 | 0.114 | 0.342 |
|  |  |  |  | rs10906609 | 14172745 | 0.082 | 0.292 |
|  |  |  |  | **rs752962** | 14177928 | 0.144 | 0.292 |
|  |  |  |  | rs12217676 | 14181851 | 0.081 | 0.325 |
|  |  |  |  | rs7914647 | 14190039 | 0.093 | 0.367 |
| 9 | 8 | *OR8U8* | 11 | rs2128133 | 56122057 | 0.097 | 0.133 |
|  |  |  |  | rs661090 | 56209204 | 0.116 | 0.125 |
|  |  |  |  | rs7939886 | 56242261 | 0.081 | 0.100 |
|  |  |  |  | rs676901 | 56295143 | 0.098 | 0.208 |
|  |  |  |  | rs11607910 | 56334457 | 0.087 | 0.158 |
|  |  |  |  | rs1945282 | 56371633 | 0.089 | 0.208 |
|  |  |  |  | **rs1397056** | 56421197 | 0.116 | 0.267 |
|  |  |  |  | rs11228715 | 56439065 | 0.083 | 0.383 |
|  |  |  |  | rs10896511 | 56462593 | 0.121 | 0.250 |
| 10 | 7 | *ARHGAP24* | 4 | rs17410455 | 86815663 | 0.098 | 0.150 |
|  |  |  |  | rs35900112 | 86817718 | 0.090 | 0.492 |
|  |  |  |  | rs346509 | 86835805 | 0.086 | 0.292 |
|  |  |  |  | rs346500 | 86853558 | 0.089 | 0.500 |
|  |  |  |  | **rs346501** | 86854664 | 0.120 | 0.500 |
|  |  |  |  | rs346507 | 86861906 | 0.109 | 0.342 |
|  |  |  |  | rs346482 | 86876674 | 0.095 | 0.442 |
|  |  |  |  | rs346473 | 86901621 | 0.086 | 0.450 |
|  |  |  |  | rs11724681 | 86912951 | 0.086 | 0.325 |
|  |  |  |  | rs2589504 | 86917655 | 0.080 | 0.325 |
|  |  |  |  | rs2164537 | 86927197 | 0.095 | 0.325 |
| 11 | 7 | *CNTLN* | 9 | rs10810729 | 17125742 | 0.100 | 0.125 |
|  |  |  |  | **rs411167** | 17130360 | 0.081 | 0.425 |
|  |  |  |  | rs3808795 | 17273731 | 0.086 | 0.217 |
|  |  |  |  | rs12379655 | 17273926 | 0.090 | 0.217 |
|  |  |  |  | rs4405013 | 17325122 | 0.093 | 0.217 |
|  |  |  |  | rs4961551 | 17367673 | 0.088 | 0.183 |
|  |  |  |  | rs7019482 | 17413556 | 0.083 | 0.325 |
| 12 | 7 | *LPPR1* | 9 | rs2480733 | 103769414 | 0.090 | 0.383 |
|  |  |  |  | rs481960 | 103785703 | 0.091 | 0.417 |
|  |  |  |  | **rs516081** | 103787135 | 0.104 | 0.375 |
|  |  |  |  | rs2254413 | 103838487 | 0.080 | 0.383 |
|  |  |  |  | rs2987754 | 103860822 | 0.082 | 0.475 |
|  |  |  |  | rs2567312 | 104032801 | 0.086 | 0.300 |
|  |  |  |  | rs13289023 | 104034641 | 0.088 | 0.392 |
|  |  |  |  | rs1341769 | 104064348 | 0.087 | 0.367 |
| 13 | 7 | *DLG2* | 11 | rs11233848 | 83662405 | 0.085 | 0.142 |
|  |  |  |  | rs1943714 | 84558671 | 0.081 | 0.483 |
|  |  |  |  | rs624493 | 84722252 | 0.088 | 0.142 |
|  |  |  |  | **rs612389** | 84724185 | 0.127 | 0.142 |
|  |  |  |  | rs483220 | 84750686 | 0.088 | 0.133 |
|  |  |  |  | rs10898380 | 84920320 | 0.081 | 0.042 |
|  |  |  |  | rs10501590 | 84943077 | 0.082 | 0.100 |
|  |  |  |  | rs551940 | 85046246 | 0.090 | 0.083 |
|  |  |  |  | rs286526 | 85206355 | 0.113 | 0.092 |
| 14 | 6 | *PDE1C* | 7 | rs10257095 | 31762127 | 0.099 | 0.225 |
|  |  |  |  | rs6945316 | 31799663 | 0.089 | 0.475 |
|  |  |  |  | **rs6945688** | 31799990 | 0.114 | 0.475 |
|  |  |  |  | rs11981972 | 31800878 | 0.091 | 0.358 |
|  |  |  |  | rs10275897 | 31812263 | 0.116 | 0.283 |
|  |  |  |  | rs12154811 | 31812676 | 0.083 | 0.283 |
|  |  |  |  | rs28688395 | 31812761 | 0.082 | 0.358 |
|  |  |  |  | rs6961705 | 31813385 | 0.085 | 0.358 |
|  |  |  |  | rs12701142 | 31874875 | 0.089 | 0.258 |
|  |  |  |  | rs2191877 | 31883140 | 0.085 | 0.093 |
| 15 | 6 | *PCDH15* | 10 | rs7070060 | 56196319 | 0.096 | 0.458 |
|  |  |  |  | rs11597868 | 56197903 | 0.090 | 0.458 |
|  |  |  |  | rs12219365 | 56977265 | 0.081 | 0.267 |
|  |  |  |  | rs1822763 | 56980468 | 0.086 | 0.425 |
|  |  |  |  | rs2799039 | 56994447 | 0.082 | 0.425 |
|  |  |  |  | rs2799038 | 56995810 | 0.100 | 0.425 |
|  |  |  |  | **rs10825483** | 57007182 | 0.110 | 0.417 |
| 16 | 6 | *TLL2* | 10 | rs11188739 | 98135631 | 0.094 | 0.358 |
|  |  |  |  | rs2093558 | 98140048 | 0.080 | 0.492 |
|  |  |  |  | rs7919721 | 98158494 | 0.081 | 0.475 |
|  |  |  |  | **rs3789950** | 98162791 | 0.101 | 0.417 |
|  |  |  |  | rs7900011 | 98167968 | 0.090 | 0.492 |
|  |  |  |  | rs730179 | 98171325 | 0.096 | 0.400 |
| 17 | 6 | *TMEM132D* | 12 | rs155370 | 130077096 | 0.095 | 0.425 |
|  |  |  |  | **rs155681** | 130077125 | 0.099 | 0.500 |
|  |  |  |  | rs155680 | 130077390 | 0.084 | 0.433 |
|  |  |  |  | rs12821839 | 130095990 | 0.094 | 0.192 |
|  |  |  |  | rs12826613 | 130101770 | 0.084 | 0.322 |
|  |  |  |  | rs264514 | 130167749 | 0.080 | 0.267 |
|  |  |  |  | rs1376795 | 130280270 | 0.081 | 0.308 |
| 18 | 6 | *MACROD2* | 20 | rs41328645 | 14428703 | 0.092 | 0.225 |
|  |  |  |  | rs13036305 | 14479373 | 0.115 | 0.233 |
|  |  |  |  | rs2423801 | 14507610 | 0.085 | 0.225 |
|  |  |  |  | rs2423856 | 14917186 | 0.089 | 0.075 |
|  |  |  |  | **rs6110533** | 15122578 | 0.106 | 0.375 |
|  |  |  |  | rs459874 | 15139547 | 0.098 | 0.375 |
|  |  |  |  | rs6135644 | 16008011 | 0.087 | 0.158 |
|  |  |  |  | rs7262019 | 16168007 | 0.091 | 0.167 |
| 19 | 5 | *FPGT-TNNI3K* | 1 | rs10493539 | 74715577 | 0.081 | 0.308 |
|  |  |  |  | **rs12027334** | 74842787 | 0.100 | 0.333 |
|  |  |  |  | rs41323947 | 74844603 | 0.085 | 0.108 |
|  |  |  |  | rs6693806 | 74861767 | 0.093 | 0.283 |
|  |  |  |  | rs7551507 | 74995225 | 0.083 | 0.425 |
| 20 | 5 | *SLC6A1* | 3 | rs11718132 | 11045020 | 0.081 | 0.208 |
|  |  |  |  | rs11710497 | 11045896 | 0.098 | 0.167 |
|  |  |  |  | rs6778281 | 11053517 | 0.087 | 0.092 |
|  |  |  |  | **rs11708202** | 11066055 | 0.092 | 0.208 |
|  |  |  |  | rs9822125 | 11066876 | 0.080 | 0.117 |
| 21 | 5 | *EPHB1* | 3 | rs1982353 | 134381229 | 0.089 | 0.458 |
|  |  |  |  | rs17765097 | 134816417 | 0.101 | 0.333 |
|  |  |  |  | rs9821105 | 134818733 | 0.083 | 0.325 |
|  |  |  |  | rs7373984 | 134820091 | 0.085 | 0.392 |
|  |  |  |  | **rs7374822** | 134820184 | 0.137 | 0.400 |
|  |  |  |  | rs2140766 | 134843286 | 0.084 | 0.333 |
| 22 | 5 | *LOC285419* | 4 | rs1347188 | 124743259 | 0.082 | 0.200 |
|  |  |  |  | rs3113389 | 124766080 | 0.094 | 0.342 |
|  |  |  |  | **rs3097903** | 124766331 | 0.135 | 0.342 |
|  |  |  |  | rs10033637 | 124833307 | 0.086 | 0.333 |
|  |  |  |  | rs3113376 | 124833917 | 0.105 | 0.392 |
|  |  |  |  | rs3113377 | 124834671 | 0.085 | 0.400 |
|  |  |  |  | rs6838706 | 124851510 | 0.099 | 0.458 |
| 23 | 5 | *NPSR1-AS1* | 7 | rs9639694 | 34538922 | 0.085 | 0.458 |
|  |  |  |  | **rs2041301** | 34540560 | 0.093 | 0.467 |
|  |  |  |  | rs6950303 | 34587196 | 0.080 | 0.100 |
|  |  |  |  | rs17169864 | 34610590 | 0.087 | 0.100 |
|  |  |  |  | rs2530550 | 34698251 | 0.081 | 0.317 |
|  |  |  |  | rs323925 | 34730255 | 0.081 | 0.133 |
| 24 | 5 | *CPED1* | 7 | rs12176609 | 120711288 | 0.086 | 0.383 |
|  |  |  |  | rs10261386 | 120711855 | 0.083 | 0.417 |
|  |  |  |  | rs10953925 | 120715154 | 0.096 | 0.467 |
|  |  |  |  | **rs12666340** | 120716152 | 0.128 | 0.467 |
|  |  |  |  | rs2110280 | 120718419 | 0.083 | 0.475 |
|  |  |  |  | rs6466767 | 120731577 | 0.103 | 0.325 |
|  |  |  |  | rs798933 | 120736615 | 0.086 | 0.325 |
|  |  |  |  | rs6954757 | 120743182 | 0.107 | 0.325 |
| 25 | 5 | *CNTNAP2* | 7 | rs2620439 | 146993413 | 0.089 | 0.450 |
|  |  |  |  | rs12703906 | 147040778 | 0.088 | 0.458 |
|  |  |  |  | rs17412444 | 147041372 | 0.082 | 0.358 |
|  |  |  |  | rs4621717 | 147131270 | 0.083 | 0.242 |
|  |  |  |  | rs1528529 | 147132505 | 0.086 | 0.242 |
|  |  |  |  | **rs10242076** | 147155515 | 0.104 | 0.342 |
|  |  |  |  | rs2022226 | 147157421 | 0.081 | 0.358 |
|  |  |  |  | rs11981369 | 147167173 | 0.094 | 0.242 |
| 26 | 5 | *PALM2-AKAP2* | 9 | rs7028076 | 112817182 | 0.099 | 0.242 |
|  |  |  |  | rs2017392 | 112820620 | 0.092 | 0.325 |
|  |  |  |  | rs2017219 | 112820742 | 0.094 | 0.325 |
|  |  |  |  | rs5942539 | 112826521 | 0.105 | 0.400 |
|  |  |  |  | **rs487013** | 112831877 | 0.127 | 0.450 |
| 27 | 5 | *LUZP2* | 11 | rs7932020 | 24513113 | 0.096 | 0.083 |
|  |  |  |  | rs9667545 | 24520669 | 0.113 | 0.100 |
|  |  |  |  | **rs4922683** | 24535089 | 0.158 | 0.133 |
|  |  |  |  | rs4923206 | 24751859 | 0.081 | 0.108 |
|  |  |  |  | rs10767269 | 24908161 | 0.084 | 0.400 |
| 28 | 5 | *C11orf63* | 11 | rs10892928 | 122801099 | 0.081 | 0.258 |
|  |  |  |  | rs4293126 | 122804133 | 0.085 | 0.483 |
|  |  |  |  | rs4502006 | 122805910 | 0.094 | 0.322 |
|  |  |  |  | rs7110039 | 122834001 | 0.090 | 0.458 |
|  |  |  |  | rs7124683 | 122834036 | 0.084 | 0.458 |
|  |  |  |  | rs930983 | 122834414 | 0.095 | 0.458 |
| 29 | 5 | *NPAS3* | 14 | **rs17491067** | 33838909 | 0.092 | 0.300 |
|  |  |  |  | rs10483449 | 33980462 | 0.091 | 0.142 |
|  |  |  |  | rs1958550 | 34065521 | 0.083 | 0.100 |
|  |  |  |  | rs13379162 | 34166988 | 0.082 | 0.258 |
|  |  |  |  | rs10498321 | 34194231 | 0.102 | 0.167 |
| 30 | 5 | *PTPRM* | 18 | rs385769 | 7800480 | 0.094 | 0.467 |
|  |  |  |  | rs7239136 | 7829163 | 0.081 | 0.400 |
|  |  |  |  | rs7235300 | 7837860 | 0.080 | 0.408 |
|  |  |  |  | rs9956121 | 8424547 | 0.082 | 0.233 |
|  |  |  |  | rs17494870 | 8471372 | 0.081 | 0.467 |
| 31 | 5 | *DCC* | 18 | rs3862681 | 49930690 | 0.085 | 0.142 |
|  |  |  |  | rs12608115 | 50227036 | 0.085 | 0.483 |
|  |  |  |  | rs4468701 | 50611280 | 0.088 | 0.483 |
|  |  |  |  | rs4459619 | 50620708 | 0.081 | 0.500 |
|  |  |  |  | rs4278818 | 50692978 | 0.088 | 0.450 |
|  |  |  |  | rs4277413 | 50698926 | 0.084 | 0.450 |
| 32 | 5 | *SYN3* | 22 | rs2105865 | 32950053 | 0.081 | 0.217 |
|  |  |  |  | **rs2413151** | 33167869 | 0.110 | 0.283 |
|  |  |  |  | rs4992163 | 33170856 | 0.081 | 0.333 |
|  |  |  |  | rs978808 | 33171931 | 0.108 | 0.333 |
|  |  |  |  | rs5754395 | 33444278 | 0.103 | 0.167 |
| 33 | 11 | *-* | 11 | **rs4434965** | 90458183 | 0.104 | 0.458 |
|  |  |  |  | rs2187536 | 90463275 | 0.091 | 0.408 |
|  |  |  |  | rs2226919 | 90464441 | 0.094 | 0.458 |
|  |  |  |  | rs4477404 | 90465406 | 0.087 | 0.458 |
|  |  |  |  | rs7937113 | 90466385 | 0.083 | 0.408 |
|  |  |  |  | rs10734134 | 90472836 | 0.087 | 0.450 |
|  |  |  |  | rs10734135 | 90472975 | 0.092 | 0.442 |
|  |  |  |  | rs4451691 | 90473356 | 0.086 | 0.408 |
|  |  |  |  | rs4500454 | 90474537 | 0.102 | 0.458 |
|  |  |  |  | rs1894136 | 90498019 | 0.093 | 0.458 |
|  |  |  |  | rs1894135 | 90498227 | 0.095 | 0.458 |
|  |  |  |  | rs10741370 | 90500110 | 0.081 | 0.458 |
|  |  |  |  | rs10741371 | 90500153 | 0.093 | 0.408 |
|  |  |  |  | rs10830574 | 90507561 | 0.084 | 0.408 |
|  |  |  |  | rs7127256 | 90525490 | 0.093 | 0.458 |
|  |  |  |  | rs7952309 | 90526114 | 0.089 | 0.450 |
|  |  |  |  | rs7952437 | 90526222 | 0.103 | 0.392 |
|  |  |  |  | rs12291122 | 90531578 | 0.098 | 0.450 |
|  |  |  |  | rs10741376 | 90538099 | 0.085 | 0.392 |
| 34 | 10 | *-* | 13 | rs1935108 | 70894396 | 0.083 | 0.458 |
|  |  |  |  | rs6562626 | 70904729 | 0.100 | 0.407 |
|  |  |  |  | rs9542286 | 70908829 | 0.109 | 0.475 |
|  |  |  |  | rs1014745 | 70909972 | 0.100 | 0.383 |
|  |  |  |  | rs4884886 | 70924474 | 0.100 | 0.383 |
|  |  |  |  | rs9599636 | 70925438 | 0.083 | 0.375 |
|  |  |  |  | rs9599640 | 70926073 | 0.086 | 0.475 |
|  |  |  |  | rs9599641 | 70926099 | 0.090 | 0.383 |
|  |  |  |  | rs9599643 | 70927978 | 0.105 | 0.433 |
|  |  |  |  | rs9599645 | 70928041 | 0.091 | 0.433 |
|  |  |  |  | rs9599646 | 70928075 | 0.093 | 0.433 |
|  |  |  |  | rs1930872 | 70935769 | 0.109 | 0.375 |
|  |  |  |  | rs1930873 | 70935786 | 0.088 | 0.375 |
|  |  |  |  | rs2325287 | 70938498 | 0.082 | 0.467 |
|  |  |  |  | **rs4883870** | 70940325 | 0.131 | 0.483 |
|  |  |  |  | rs12870439 | 70947913 | 0.131 | 0.283 |
|  |  |  |  | rs12859922 | 70951062 | 0.101 | 0.242 |
|  |  |  |  | rs12875581 | 70957534 | 0.098 | 0.242 |
|  |  |  |  | rs12875114 | 70987292 | 0.089 | 0.233 |
| 35 | 7 | *-* | 5 | rs7727315 | 117084225 | 0.094 | 0.475 |
|  |  |  |  | rs7727755 | 117084701 | 0.094 | 0.475 |
|  |  |  |  | rs4482937 | 117087858 | 0.086 | 0.283 |
|  |  |  |  | rs12188743 | 117095332 | 0.102 | 0.242 |
|  |  |  |  | rs4304103 | 117095928 | 0.105 | 0.483 |
|  |  |  |  | **rs13188604** | 117116009 | 0.115 | 0.492 |
|  |  |  |  | rs12110276 | 117116443 | 0.097 | 0.492 |
|  |  |  |  | rs4547959 | 117127287 | 0.112 | 0.483 |
|  |  |  |  | rs4244395 | 117131770 | 0.092 | 0.467 |
|  |  |  |  | rs7727383 | 117133047 | 0.085 | 0.258 |
|  |  |  |  | rs13158826 | 117142587 | 0.081 | 0.242 |
|  |  |  |  | rs4354082 | 117147798 | 0.085 | 0.425 |
|  |  |  |  | rs11948707 | 117147971 | 0.103 | 0.425 |
| 36 | 7 | *-* | 14 | **rs11847260** | 95281246 | 0.083 | 0.433 |
|  |  |  |  | rs4418997 | 95281941 | 0.090 | 0.300 |
|  |  |  |  | rs7154643 | 95284237 | 0.099 | 0.342 |
|  |  |  |  | rs11626503 | 95343149 | 0.093 | 0.267 |
|  |  |  |  | rs1741232 | 95359993 | 0.085 | 0.408 |
|  |  |  |  | rs1741237 | 95361346 | 0.083 | 0.300 |
|  |  |  |  | rs10484048 | 95362211 | 0.119 | 0.142 |
| 37 | 6 | *-* | 6 | rs9384701 | 109557542 | 0.090 | 0.242 |
|  |  |  |  | rs932223 | 109586656 | 0.092 | 0.408 |
|  |  |  |  | rs9384703 | 109586803 | 0.084 | 0.342 |
|  |  |  |  | rs9384704 | 109587209 | 0.093 | 0.342 |
|  |  |  |  | rs13210693 | 109598964 | 0.090 | 0.467 |
|  |  |  |  | rs9386788 | 109600523 | 0.095 | 0.467 |
|  |  |  |  | rs7755970 | 109602359 | 0.093 | 0.475 |
|  |  |  |  | rs7774390 | 109602461 | 0.130 | 0.475 |
|  |  |  |  | **rs9487033** | 109605388 | 0.135 | 0.417 |
|  |  |  |  | rs9487034 | 109605899 | 0.107 | 0.417 |
|  |  |  |  | rs9400269 | 109606065 | 0.110 | 0.417 |
|  |  |  |  | rs9386790 | 109608460 | 0.119 | 0.417 |
|  |  |  |  | rs7772602 | 109608661 | 0.121 | 0.417 |
|  |  |  |  | rs1111866 | 109610846 | 0.122 | 0.417 |
|  |  |  |  | rs1111865 | 109610863 | 0.116 | 0.417 |
|  |  |  |  | rs7748918 | 109613564 | 0.091 | 0.375 |
|  |  |  |  | rs6903695 | 109631422 | 0.084 | 0.475 |
| 38 | 6 | *-* | 8 | **rs9656970** | 129681897 | 0.098 | 0.350 |
|  |  |  |  | rs13277153 | 129708542 | 0.128 | 0.258 |
|  |  |  |  | rs6470649 | 129726384 | 0.128 | 0.258 |
|  |  |  |  | rs7017882 | 129741536 | 0.087 | 0.467 |
|  |  |  |  | rs11781599 | 129745068 | 0.089 | 0.383 |
|  |  |  |  | rs6470654 | 129755460 | 0.098 | 0.267 |
|  |  |  |  | rs978589 | 129756266 | 0.084 | 0.467 |
| 39 | 6 | *-* | 10 | rs3003516 | 3004093 | 0.081 | 0.067 |
|  |  |  |  | rs12784956 | 3021935 | 0.083 | 0.092 |
|  |  |  |  | rs4881046 | 3033127 | 0.086 | 0.092 |
|  |  |  |  | rs12782126 | 3036072 | 0.084 | 0.067 |
|  |  |  |  | **rs10903913** | 3042256 | 0.137 | 0.083 |
|  |  |  |  | rs11251635 | 3049561 | 0.093 | 0.092 |
|  |  |  |  | rs11251639 | 3051112 | 0.119 | 0.092 |
|  |  |  |  | rs2279222 | 3052273 | 0.093 | 0.092 |
|  |  |  |  | rs7902402 | 3056044 | 0.098 | 0.092 |
|  |  |  |  | rs10903930 | 3058375 | 0.099 | 0.092 |
| 40 | 6 | *-* | 16 | rs12934830 | 8353467 | 0.081 | 0.217 |
|  |  |  |  | rs11077289 | 8396691 | 0.082 | 0.225 |
|  |  |  |  | rs450049 | 8407460 | 0.103 | 0.175 |
|  |  |  |  | rs7184311 | 8432593 | 0.101 | 0.158 |
|  |  |  |  | rs2122913 | 8434830 | 0.081 | 0.283 |
|  |  |  |  | **rs2122914** | 8434935 | 0.102 | 0.283 |
|  |  |  |  | rs12448378 | 8440823 | 0.093 | 0.283 |
|  |  |  |  | rs8051427 | 8445307 | 0.083 | 0.242 |
| 41 | 5 | *-* | 4 | rs7679576 | 12938795 | 0.116 | 0.392 |
|  |  |  |  | rs7659413 | 12945048 | 0.094 | 0.458 |
|  |  |  |  | rs223989 | 12972256 | 0.113 | 0.398 |
|  |  |  |  | rs1040056 | 13003758 | 0.095 | 0.383 |
|  |  |  |  | rs2673422 | 13003905 | 0.099 | 0.350 |
| 42 | 5 | *-* | 4 | rs4861947 | 182284224 | 0.084 | 0.275 |
|  |  |  |  | **rs7682400** | 182292227 | 0.138 | 0.233 |
|  |  |  |  | rs17071251 | 182300135 | 0.084 | 0.233 |
|  |  |  |  | rs4522908 | 182304873 | 0.089 | 0.267 |
|  |  |  |  | rs10035020 | 182307708 | 0.082 | 0.300 |
| 43 | 5 | *-* | 5 | rs7704762 | 2679511 | 0.089 | 0.342 |
|  |  |  |  | rs2935621 | 2685812 | 0.080 | 0.317 |
|  |  |  |  | **rs2962615** | 2689899 | 0.127 | 0.492 |
|  |  |  |  | rs2962612 | 2691343 | 0.099 | 0.458 |
|  |  |  |  | rs2935630 | 2693095 | 0.105 | 0.458 |
|  |  |  |  | rs2132868 | 2726211 | 0.088 | 0.050 |
| 44 | 5 | *-* | 5 | rs288853 | 73403021 | 0.100 | 0.200 |
|  |  |  |  | rs652095 | 73447022 | 0.089 | 0.186 |
|  |  |  |  | **rs2926721** | 73450088 | 0.084 | 0.308 |
|  |  |  |  | rs288842 | 73450544 | 0.107 | 0.275 |
|  |  |  |  | rs288841 | 73450853 | 0.102 | 0.275 |
|  |  |  |  | rs158807 | 73474602 | 0.082 | 0.208 |
| 45 | 5 | *-* | 6 | **rs1495980** | 19386230 | 0.102 | 0.317 |
|  |  |  |  | rs6917862 | 19399401 | 0.087 | 0.325 |
|  |  |  |  | rs9295431 | 19401814 | 0.095 | 0.300 |
|  |  |  |  | rs16882159 | 19423005 | 0.084 | 0.125 |
|  |  |  |  | rs12529358 | 19427644 | 0.115 | 0.233 |
|  |  |  |  | rs12211780 | 19436035 | 0.080 | 0.150 |
| 46 | 5 | *-* | 7 | **rs4727161** | 87988026 | 0.120 | 0.325 |
|  |  |  |  | rs733004 | 87997384 | 0.104 | 0.392 |
|  |  |  |  | rs11773152 | 88019109 | 0.109 | 0.367 |
|  |  |  |  | rs11972082 | 88021474 | 0.092 | 0.367 |
|  |  |  |  | rs10278602 | 88061671 | 0.097 | 0.408 |
|  |  |  |  | rs11761151 | 88063545 | 0.099 | 0.408 |
| 47 | 5 | *-* | 7 | rs1419600 | 125506847 | 0.084 | 0.400 |
|  |  |  |  | rs1419561 | 125574140 | 0.083 | 0.467 |
|  |  |  |  | rs1034651 | 125587606 | 0.087 | 0.425 |
|  |  |  |  | rs13245265 | 125593340 | 0.086 | 0.466 |
|  |  |  |  | rs10487429 | 125599204 | 0.091 | 0.408 |
|  |  |  |  | **rs4397338** | 125602729 | 0.098 | 0.408 |
| 48 | 5 | *-* | 9 | rs10820567 | 106696953 | 0.113 | 0.333 |
|  |  |  |  | rs7042666 | 106715594 | 0.100 | 0.125 |
|  |  |  |  | **rs10820588** | 106722204 | 0.106 | 0.450 |
|  |  |  |  | rs10820589 | 106722291 | 0.090 | 0.233 |
|  |  |  |  | rs10991016 | 106733144 | 0.098 | 0.483 |
|  |  |  |  | rs501434 | 106737655 | 0.093 | 0.483 |
| 49 | 5 | *-* | 10 | rs10508278 | 4263255 | 0.111 | 0.092 |
|  |  |  |  | rs10508279 | 4300734 | 0.137 | 0.083 |
|  |  |  |  | rs11252445 | 4330575 | 0.086 | 0.258 |
|  |  |  |  | rs11252447 | 4331726 | 0.101 | 0.212 |
|  |  |  |  | **rs9423526** | 4334578 | 0.080 | 0.267 |
| 50 | 5 | *-* | 13 | rs17055932 | 38067143 | 0.089 | 0.250 |
|  |  |  |  | **rs9547912** | 38071135 | 0.091 | 0.292 |
|  |  |  |  | rs9576286 | 38088609 | 0.094 | 0.250 |
|  |  |  |  | rs17255466 | 38100807 | 0.084 | 0.325 |
|  |  |  |  | rs10467365 | 38112413 | 0.089 | 0.333 |
| 51 | 5 | *-* | 13 | rs1491029 | 90703001 | 0.082 | 0.147 |
|  |  |  |  | rs2637485 | 90703513 | 0.083 | 0.150 |
|  |  |  |  | **rs9522832** | 90709541 | 0.101 | 0.433 |
|  |  |  |  | rs7995429 | 90716818 | 0.084 | 0.142 |
|  |  |  |  | rs2805704 | 90727635 | 0.081 | 0.342 |
|  |  |  |  | rs9515691 | 90762858 | 0.105 | 0.400 |
| 52 | 5 | *-* | 13 | rs9523154 | 91523310 | 0.093 | 0.308 |
|  |  |  |  | rs1591448 | 91523647 | 0.082 | 0.308 |
|  |  |  |  | **rs12583307** | 91536262 | 0.096 | 0.450 |
|  |  |  |  | rs272093 | 91589940 | 0.089 | 0.242 |
|  |  |  |  | rs4771828 | 91590644 | 0.086 | 0.308 |
|  |  |  |  | rs17667393 | 91607320 | 0.093 | 0.467 |
| 53 | 5 | *-* | 16 | rs9926403 | 52811866 | 0.103 | 0.425 |
|  |  |  |  | rs11644675 | 52813601 | 0.088 | 0.400 |
|  |  |  |  | rs1894898 | 52817511 | 0.112 | 0.433 |
|  |  |  |  | rs12446341 | 52818468 | 0.083 | 0.433 |
|  |  |  |  | rs7190231 | 52823984 | 0.104 | 0.433 |
|  |  |  |  | **rs8055491** | 52824235 | 0.123 | 0.433 |
|  |  |  |  | rs12599348 | 52828325 | 0.091 | 0.392 |
|  |  |  |  | rs1362417 | 52830515 | 0.115 | 0.433 |
|  |  |  |  | rs8044632 | 52830966 | 0.112 | 0.433 |
|  |  |  |  | rs2024447 | 52855494 | 0.082 | 0.417 |
|  |  |  |  | rs7202791 | 52855926 | 0.081 | 0.417 |
| 54 | 5 | *-* | 16 | rs1529925 | 64050773 | 0.104 | 0.158 |
|  |  |  |  | rs1369930 | 64067084 | 0.087 | 0.292 |
|  |  |  |  | rs12444551 | 64072486 | 0.089 | 0.175 |
|  |  |  |  | **rs2164512** | 64082446 | 0.125 | 0.225 |
|  |  |  |  | rs1369928 | 64084790 | 0.090 | 0.225 |
|  |  |  |  | rs17475408 | 64121429 | 0.101 | 0.233 |

Abbreviations - Chr.: Chromosome; bp: Base pair position (Affymetrix GenomeWideSNP_6 Annotations, release 32); |RAS_diff_|: Absolute value of the relative allele score difference between cases and controls; MAF: Minor allele frequency in the CEU HapMap samples (Affymetrix GenomeWideSNP_6 Annotations, release 32).
