# Supplementary material for: Multicentric Genome-Wide Association Study for Primary Spontaneous Pneumothorax
Source: PLoS One. 2016 May 20;11(5):e0156103. doi: 10.1371/journal.pone.0156103 (PMC4874577; doi:10.1371/journal.pone.0156103)
Supplement: S4 Table — The markers are sorted by increasing P-value and the top 49 SNPs highlighted in bold were selected for technical validation through this approach. (DOCX) [file pone.0156103.s006.docx]

**S4 Table. Top 100 SNPs in the PSP GWAS discovery phase according to the combined Z-test.** The markers are sorted by increasing *P*-value and the top 49 SNPs highlighted in bold were selected for technical validation through this approach.

| **Affymetrix SNP ID** | **SNP** | **Chr.** | **Position (bp)** | **Gene** | **MAF** | ***P*-value** |
| --- | --- | --- | --- | --- | --- | --- |
| SNP_A-1953716 | **rs4922683** | 11 | 24535089 | *LUZP2* | 0.158 | 6.28E-04 |
| SNP_A-4303960 | **rs7741604** | 6 | 20731524 | *CDKAL1* | 0.162 | 7.29E-04 |
| SNP_A-1988278 | **rs287903** | 6 | 157164550 | *ARID1B* | 0.159 | 8.56E-04 |
| SNP_A-2129421 | **rs4423896** | 4 | 78340170 | *-* | 0.157 | 9.72E-04 |
| SNP_A-2172641 | **rs6902892** | 6 | 109627601 | *CCDC162* | 0.156 | 1.25E-03 |
| SNP_A-8284151 | **rs10504160** | 8 | 55013096 | *LYPLA1* | 0.191 | 1.52E-03 |
| SNP_A-4254240 | **rs139167** | 22 | 44598692 | *PARVG* | 0.140 | 1.60E-03 |
| SNP_A-1906024 | **rs2101167** | 3 | 156691408 | *LEKR1* | 0.167 | 1.87E-03 |
| SNP_A-1926346 | **rs2545886** | 16 | 21029197 | *DNAH3* | 0.148 | 2.00E-03 |
| SNP_A-1992858 | **rs10508279** | 10 | 4300734 | *-* | 0.137 | 2.04E-03 |
| SNP_A-8370747 | **rs17671063** | 7 | 68381395 | *-* | 0.154 | 2.04E-03 |
| SNP_A-8591019 | **rs7241671** | 18 | 72764169 | *ZNF407* | 0.136 | 2.16E-03 |
| SNP_A-1845593 | **rs4602358** | 3 | 42303037 | *CCK* | 0.149 | 2.33E-03 |
| SNP_A-1923579 | **rs7767391** | 6 | 20725240 | *CDKAL1* | 0.147 | 2.33E-03 |
| SNP_A-2155416 | **rs16855688** | 3 | 170772678 | *-* | 0.125 | 2.46E-03 |
| SNP_A-8643425 | **rs1131535** | 3 | 172224075 | *TNFSF10* | 0.150 | 2.60E-03 |
| SNP_A-4207170 | **rs2919427** | 16 | 30669091 | *-* | 0.162 | 2.66E-03 |
| SNP_A-8463662 | **rs1526483** | 7 | 83506643 | *-* | 0.132 | 2.67E-03 |
| SNP_A-8385981 | **rs752962** | 10 | 14177928 | *FRMD4A* | 0.144 | 2.68E-03 |
| SNP_A-1804934 | **rs17221652** | 10 | 1416472 | *ADARB2* | 0.143 | 2.97E-03 |
| SNP_A-8393894 | **rs459020** | 6 | 95052842 | *-* | 0.146 | 3.29E-03 |
| SNP_A-8604971 | **rs11629958** | 15 | 84147713 | *SH3GL3* | 0.141 | 3.33E-03 |
| SNP_A-2117404 | **rs7911954** | 10 | 2160519 | *-* | 0.138 | 3.39E-03 |
| SNP_A-2037621 | **rs236715** | 20 | 57697095 | *-* | 0.135 | 3.48E-03 |
| SNP_A-4296596 | **rs17133680** | 10 | 4874019 | *AKR1E2* | 0.109 | 3.58E-03 |
| SNP_A-8530724 | **rs1962137** | 11 | 89990909 | *-* | 0.133 | 3.69E-03 |
| SNP_A-1862608 | **rs7831961** | 8 | 135507060 | *ZFAT* | 0.146 | 3.87E-03 |
| SNP_A-2144398 | **rs10966315** | 9 | 24467996 | *-* | 0.127 | 3.99E-03 |
| SNP_A-8670099 | **rs6466365** | 7 | 110577992 | *IMMP2L* | 0.153 | 4.12E-03 |
| SNP_A-8367558 | **rs6531429** | 4 | 36275383 | *-* | 0.145 | 4.20E-03 |
| SNP_A-8475662 | **rs1333199** | 10 | 2185200 | *-* | 0.141 | 4.67E-03 |
| SNP_A-1852386 | **rs10222715** | 4 | 57614160 | *-* | 0.142 | 4.71E-03 |
| SNP_A-4275369 | **rs9487033** | 6 | 109605388 | *-* | 0.135 | 4.74E-03 |
| SNP_A-1950562 | **rs230833** | 5 | 56915585 | *-* | 0.122 | 4.87E-03 |
| SNP_A-2036636 | **rs4457905** | 14 | 30233422 | *PRKD1* | 0.133 | 4.95E-03 |
| SNP_A-2035616 | **rs7463038** | 8 | 38587043 | *TACC1* | 0.124 | 4.97E-03 |
| SNP_A-1876776 | **rs287916** | 6 | 157159008 | *ARID1B* | 0.130 | 5.04E-03 |
| SNP_A-2047275 | **rs10942788** | 5 | 75957603 | *IQGAP2* | 0.141 | 5.11E-03 |
| SNP_A-1929490 | **rs6983560** | 8 | 135584553 | *ZFAT* | 0.132 | 5.19E-03 |
| SNP_A-8283740 | **rs1353318** | 8 | 15338089 | *-* | 0.127 | 5.34E-03 |
| SNP_A-8360108 | **rs8083684** | 18 | 44947391 | *-* | 0.116 | 5.44E-03 |
| SNP_A-8285645 | **rs10491529** | 9 | 122932725 | *-* | 0.130 | 5.72E-03 |
| SNP_A-8423678 | **rs1525833** | 7 | 46069653 | *-* | 0.127 | 5.98E-03 |
| SNP_A-8360659 | **rs4377469** | 3 | 42303074 | *CCK* | 0.123 | 6.08E-03 |
| SNP_A-2151292 | **rs7682400** | 4 | 182292227 | *-* | 0.138 | 6.36E-03 |
| SNP_A-2249128 | **rs10484048** | 14 | 95362211 | *-* | 0.119 | 6.39E-03 |
| SNP_A-8509416 | **rs612389** | 11 | 84724185 | *DLG2* | 0.127 | 6.41E-03 |
| SNP_A-1882930 | **rs488940** | 11 | 55261905 | *-* | 0.132 | 6.70E-03 |
| SNP_A-1814946 | **rs10903913** | 10 | 3042256 | *-* | 0.137 | 6.79E-03 |
| SNP_A-8564699 | rs7020926 | 9 | 112329792 | *-* | 0.115 | 6.83E-03 |
| SNP_A-2005775 | rs4605265 | 18 | 67255632 | *DOK6* | 0.122 | 6.94E-03 |
| SNP_A-8518753 | rs4883870 | 13 | 70940325 | *-* | 0.131 | 6.99E-03 |
| SNP_A-2125156 | rs133561 | 22 | 48639063 | *-* | 0.125 | 7.07E-03 |
| SNP_A-4264752 | rs10491237 | 5 | 10912382 | *-* | 0.119 | 7.19E-03 |
| SNP_A-1874570 | rs7374822 | 3 | 134820184 | *EPHB1* | 0.137 | 7.25E-03 |
| SNP_A-8427831 | rs155123 | 2 | 182312272 | *-* | 0.131 | 7.35E-03 |
| SNP_A-1968607 | rs10485620 | 20 | 50565168 | *-* | 0.124 | 7.39E-03 |
| SNP_A-2112567 | rs723436 | 13 | 22331539 | *-* | 0.135 | 7.59E-03 |
| SNP_A-2170991 | rs3097903 | 4 | 124766331 | *LOC285419* | 0.135 | 7.79E-03 |
| SNP_A-8562522 | rs10956847 | 8 | 93498901 | *-* | 0.132 | 7.96E-03 |
| SNP_A-1995274 | rs6988000 | 8 | 135559534 | *ZFAT* | 0.112 | 7.98E-03 |
| SNP_A-4206508 | rs12870439 | 13 | 70947913 | *-* | 0.131 | 8.48E-03 |
| SNP_A-2066116 | rs12792701 | 11 | 28988343 | *-* | 0.136 | 8.53E-03 |
| SNP_A-4293603 | rs2971955 | 7 | 133665939 | *EXOC4* | 0.132 | 8.58E-03 |
| SNP_A-8640336 | rs1547299 | 13 | 71001032 | *-* | 0.128 | 8.65E-03 |
| SNP_A-1936662 | rs4602638 | 5 | 31122961 | *-* | 0.134 | 8.75E-03 |
| SNP_A-2063783 | rs6734558 | 2 | 139010773 | *-* | 0.116 | 8.76E-03 |
| SNP_A-4289018 | rs7774390 | 6 | 109602461 | *-* | 0.130 | 8.79E-03 |
| SNP_A-2198642 | rs931028 | 10 | 71373767 | *-* | 0.120 | 8.91E-03 |
| SNP_A-4207233 | rs10088760 | 8 | 112341321 | *-* | 0.134 | 8.93E-03 |
| SNP_A-8292508 | rs4978396 | 9 | 112269480 | *-* | 0.130 | 9.14E-03 |
| SNP_A-4225651 | rs6064503 | 20 | 37026379 | *-* | 0.129 | 9.23E-03 |
| SNP_A-2271057 | rs4354342 | 8 | 135273013 | *-* | 0.129 | 9.30E-03 |
| SNP_A-4255855 | rs36759 | 5 | 97901942 | *-* | 0.110 | 9.37E-03 |
| SNP_A-8603283 | rs10498321 | 14 | 34194231 | *NPAS3* | 0.102 | 9.40E-03 |
| SNP_A-8665296 | rs428751 | 5 | 101465106 | *-* | 0.129 | 9.57E-03 |
| SNP_A-8298577 | rs4733649 | 8 | 129798114 | *-* | 0.135 | 9.66E-03 |
| SNP_A-8589391 | rs4669423 | 2 | 9839447 | *-* | 0.106 | 9.71E-03 |
| SNP_A-1826262 | rs2144862 | 10 | 52260398 | *SGMS1* | 0.126 | 9.74E-03 |
| SNP_A-8685522 | rs6881724 | 5 | 97846394 | *-* | 0.133 | 9.77E-03 |
| SNP_A-2054267 | rs733254 | 8 | 135638632 | *ZFAT* | 0.128 | 9.78E-03 |
| SNP_A-8467764 | rs29035 | 18 | 9996143 | *-* | 0.127 | 9.82E-03 |
| SNP_A-8318599 | rs2228211 | 6 | 12122773 | *HIVEP1* | 0.129 | 1.00E-02 |
| SNP_A-8621763 | rs941704 | 14 | 68005110 | *PLEKHH1* | 0.112 | 1.01E-02 |
| SNP_A-8401880 | rs4429607 | 3 | 189454251 | *TP63* | 0.115 | 1.02E-02 |
| SNP_A-1828740 | rs41401345 | 5 | 168299197 | *SLIT3* | 0.114 | 1.03E-02 |
| SNP_A-4195069 | rs10896511 | 11 | 56462593 | *OR8U8* | 0.121 | 1.03E-02 |
| SNP_A-8311750 | rs2058487 | 9 | 122945873 | *-* | 0.132 | 1.03E-02 |
| SNP_A-1960639 | rs6663840 | 1 | 3743319 | *KIAA0562* | 0.122 | 1.03E-02 |
| SNP_A-8368745 | rs6887429 | 5 | 117212923 | *-* | 0.120 | 1.04E-02 |
| SNP_A-2107533 | rs16906858 | 12 | 30983129 | *-* | 0.132 | 1.04E-02 |
| SNP_A-2026110 | rs2962615 | 5 | 2689899 | *-* | 0.127 | 1.05E-02 |
| SNP_A-2273752 | rs10874963 | 1 | 96343561 | *-* | 0.122 | 1.05E-02 |
| SNP_A-8402116 | rs487013 | 9 | 112831877 | *AKAP2* | 0.127 | 1.08E-02 |
| SNP_A-4302617 | rs11742607 | 5 | 172966051 | *-* | 0.192 | 1.10E-02 |
| SNP_A-1924975 | rs12449855 | 17 | 46485603 | *SKAP1* | 0.042 | 1.10E-02 |
| SNP_A-4286781 | rs11780632 | 8 | 135606615 | *ZFAT* | 0.183 | 1.10E-02 |
| SNP_A-1795987 | rs1290558 | 1 | 118065841 | *MAN1A2* | 0.292 | 1.11E-02 |
| SNP_A-8623444 | rs6466115 | 7 | 106111672 | - | 0.050 | 1.12E-02 |
| SNP_A-2258793 | rs17562236 | 1 | 75374025 | - | 0.283 | 1.12E-02 |

Abbreviations - Chr.: Chromosome; bp: Base pair position (Affymetrix GenomeWideSNP_6 Annotations, release 32); MAF: Minor allele frequency in the CEU HapMap samples (Affymetrix GenomeWideSNP_6 Annotations, release 32).
