# Supplementary material for: Multicentric Genome-Wide Association Study for Primary Spontaneous Pneumothorax
Source: PLoS One. 2016 May 20;11(5):e0156103. doi: 10.1371/journal.pone.0156103 (PMC4874577; doi:10.1371/journal.pone.0156103)
Supplement: S5 Table — The SNPs selected by the |RASdiff| method are listed first (48 SNPs), followed by those selected through the cluster method (total of 54 SNPs, 9 of which have already been selected by the |RASdiff| strategy) and finally the combined Z-test (total of 49 SNPs, 37 of which have already been selected by the previous two methods). For each SNP, the |RASdiff|, LD score/Cluster ID and P-value for a given SNP are only indicated if the SNP passed the selection threshold in the respective method. (DOCX) [file pone.0156103.s007.docx]

**S5 Table. 101 SNPs from the GWAS discovery phase prioritized for technical validation using three approaches (|RASdiff|, cluster method and combined Z-test).** The SNPs selected by the |RAS_diff_| method are listed first (48 SNPs), followed by those selected through the cluster method (total of 54 SNPs, 9 of which have already been selected by the |RAS_diff_| strategy) and finally the combined Z-test (total of 49 SNPs, 37 of which have already been selected by the previous two methods). For each SNP, the |RAS_diff_|, LD score/Cluster ID and *P*-value for a given SNP are only indicated if the SNP passed the selection threshold in the respective method.

| **SNP** | **Chr.** | **Gene** | **\|RAS_diff_\|** | **LD score** | **Cluster ID** | ***P*-value** |
| --- | --- | --- | --- | --- | --- | --- |
| rs10504160 | 8 | *LYPLA1* | 0.191 | - | - | 1.52E-03 |
| rs2101167 | 3 | *LEKR1* | 0.167 | - | - | 1.87E-03 |
| rs7741604 | 6 | *CDKAL1* | 0.162 | - | - | 7.29E-04 |
| rs2919427 | 16 | *-* | 0.162 | - | - | 2.66E-03 |
| rs287903 | 6 | *ARID1B* | 0.159 | - | - | 8.56E-04 |
| rs4922683 | 11 | *LUZP2* | 0.158 | 5 | 27 | 6.28E-04 |
| rs4423896 | 4 | *-* | 0.157 | - | - | 9.72E-04 |
| rs6902892 | 6 | *CCDC162* | 0.156 | - | - | 1.25E-03 |
| rs17671063 | 7 | *-* | 0.154 | - | - | 2.04E-03 |
| rs6466365 | 7 | *IMMP2L* | 0.153 | - | - | 4.12E-03 |
| rs1131535 | 3 | *TNFSF10* | 0.150 | - | - | 2.60E-03 |
| rs4602358 | 3 | *CCK* | 0.149 | - | - | 2.33E-03 |
| rs2545886 | 16 | *DNAH3* | 0.148 | - | - | 2.00E-03 |
| rs7767391 | 6 | *CDKAL1* | 0.147 | 10 | 4 | 2.33E-03 |
| rs459020 | 6 | *-* | 0.146 | - | - | 3.29E-03 |
| rs7831961 | 8 | *ZFAT* | 0.146 | 11 | 2 | 3.87E-03 |
| rs6531429 | 4 | *BC037904* | 0.145 | - | - | 4.20E-03 |
| rs752962 | 10 | *FRMD4A* | 0.144 | 9 | 8 | 2.68E-03 |
| rs17221652 | 10 | *ADARB2* | 0.143 | - | - | 2.97E-03 |
| rs10222715 | 4 | *-* | 0.142 | - | - | 4.71E-03 |
| rs10942788 | 5 | *IQGAP2* | 0.141 | - | - | 5.11E-03 |
| rs1333199 | 10 | *-* | 0.141 | - | - | 4.67E-03 |
| rs11629958 | 15 | *SH3GL3* | 0.141 | - | - | 3.33E-03 |
| rs139167 | 22 | *PARVG* | 0.140 | - | - | 1.60E-03 |
| rs7682400 | 4 | *-* | 0.138 | 5 | 42 | 6.36E-03 |
| rs7911954 | 10 | *-* | 0.138 | - | - | 3.39E-03 |
| rs7374822 | 3 | *EPHB1* | 0.137 | 5 | 21 | - |
| rs10903913 | 10 | *-* | 0.137 | 6 | 39 | 6.79E-03 |
| rs10508279 | 10 | *-* | 0.137 | - | - | 2.04E-03 |
| rs1445324 | 11 | *-* | 0.137 | - | - | - |
| rs12792701 | 11 | *-* | 0.136 | - | - | - |
| rs7241671 | 18 | *ZNF407* | 0.136 | - | - | 2.16E-03 |
| rs3097903 | 4 | *LOC285419* | 0.135 | 5 | 22 | - |
| rs9487033 | 6 | *-* | 0.135 | 6 | 37 | 4.74E-03 |
| rs4733649 | 8 | *-* | 0.135 | - | - | - |
| rs723436 | 13 | *-* | 0.135 | - | - | - |
| rs236715 | 20 | *-* | 0.135 | - | - | 3.48E-03 |
| rs4602638 | 5 | *-* | 0.134 | - | - | - |
| rs10088760 | 8 | *-* | 0.134 | - | - | - |
| rs6881724 | 5 | *-* | 0.133 | - | - | - |
| rs1962137 | 11 | *-* | 0.133 | - | - | 3.69E-03 |
| rs4457905 | 14 | *PRKD1* | 0.133 | - | - | 4.95E-03 |
| rs1526483 | 7 | *-* | 0.132 | - | - | 2.67E-03 |
| rs2971955 | 7 | *EXOC4* | 0.132 | - | - | - |
| rs10956847 | 8 | *-* | 0.132 | - | - | - |
| rs6983560 | 8 | *ZFAT* | 0.132 | - | - | 5.19E-03 |
| rs2058487 | 9 | *-* | 0.132 | - | - | - |
| rs488940 | 11 | *-* | 0.132 | - | - | 6.70E-03 |
| rs922799 | 8 | *CSMD1* | - | 21 | 1 | - |
| rs8048056 | 16 | *RBFOX1* | - | 11 | 3 | - |
| rs10808167 | 7 | *MAGI2* | - | 10 | 5 | - |
| rs13331343 | 16 | *CDH13* | - | 10 | 6 | - |
| rs436563 | 9 | *PTPRD* | - | 9 | 7 | - |
| rs1397056 | 11 | *OR8U8* | - | 8 | 9 | - |
| rs346501 | 4 | *ARHGAP24* | - | 7 | 10 | - |
| rs411167 | 9 | *CNTLN* | - | 7 | 11 | - |
| rs516081 | 9 | *LPPR1* | - | 7 | 12 | - |
| rs612389 | 11 | *DLG2* | - | 7 | 13 | 6.41E-03 |
| rs6945688 | 7 | *PDE1C* | - | 6 | 14 | - |
| rs10825483 | 10 | *PCDH15* | - | 6 | 15 | - |
| rs3789950 | 10 | *TLL2* | - | 6 | 16 | - |
| rs155681 | 12 | *TMEM132D* | - | 6 | 17 | - |
| rs6110533 | 20 | *MACROD2* | - | 6 | 18 | - |
| rs12027334 | 1 | *FPGT-TNNI3K* | - | 5 | 19 | - |
| rs11708202 | 3 | *SLC6A1* | - | 5 | 20 | - |
| rs2041301 | 7 | *NPSR1-AS1* | - | 5 | 23 | - |
| rs12666340 | 7 | *CPED1* | - | 5 | 24 | - |
| rs10242076 | 7 | *CNTNAP2* | - | 5 | 25 | - |
| rs487013 | 9 | *PALM2-AKAP2* | - | 5 | 26 | - |
| rs17491067 | 14 | *NPAS3* | - | 5 | 29 | - |
| rs2413151 | 22 | *SYN3* | - | 5 | 32 | - |
| rs4434965 | 11 | *-* | - | 11 | 33 | - |
| rs4883870 | 13 | *-* | - | 10 | 34 | - |
| rs13188604 | 5 | *-* | - | 7 | 35 | - |
| rs11847260 | 14 | *-* | - | 7 | 36 | - |
| rs9656970 | 8 | *-* | - | 6 | 38 | - |
| rs2122914 | 16 | *-* | - | 6 | 40 | - |
| rs2962615 | 5 | *-* | - | 5 | 43 | - |
| rs2926721 | 5 | *-* | - | 5 | 44 | - |
| rs1495980 | 6 | *-* | - | 5 | 45 | - |
| rs4727161 | 7 | *-* | - | 5 | 46 | - |
| rs4397338 | 7 | *-* | - | 5 | 47 | - |
| rs10820588 | 9 | *-* | - | 5 | 48 | - |
| rs9423526 | 10 | *-* | - | 5 | 49 | - |
| rs9547912 | 13 | *-* | - | 5 | 50 | - |
| rs9522832 | 13 | *-* | - | 5 | 51 | - |
| rs12583307 | 13 | *-* | - | 5 | 52 | - |
| rs8055491 | 16 | *-* | - | 5 | 53 | - |
| rs2164512 | 16 | *-* | - | 5 | 54 | - |
| rs16855688 | 3 | *-* | - | - | - | 2.46E-03 |
| rs17133680 | 10 | *AKR1E2* | - | - | - | 3.58E-03 |
| rs10966315 | 9 | *-* | - | - | - | 3.99E-03 |
| rs230833 | 5 | *-* | - | - | - | 4.87E-03 |
| rs7463038 | 8 | *TACC1* | - | - | - | 4.97E-03 |
| rs287916 | 6 | *ARID1B* | - | - | - | 5.04E-03 |
| rs1353318 | 8 | *-* | - | - | - | 5.34E-03 |
| rs8083684 | 18 | *BC04086* | - | - | - | 5.44E-03 |
| rs10491529 | 9 | *-* | - | - | - | 5.72E-03 |
| rs1525833 | 7 | *-* | - | - | - | 5.98E-03 |
| rs4377469 | 3 | *CCK* | - | - | - | 6.08E-03 |
| rs10484048 | 14 | *-* | - | - | - | 6.39E-03 |

Abbreviations - Chr.: Chromosome; bp: Base pair position (Affymetrix GenomeWideSNP_6 Annotations, release 32); |RASdiff|: Absolute value of the relative allele score difference between cases and controls.
