# Supplementary material for: Multicentric Genome-Wide Association Study for Primary Spontaneous Pneumothorax
Source: PLoS One. 2016 May 20;11(5):e0156103. doi: 10.1371/journal.pone.0156103 (PMC4874577; doi:10.1371/journal.pone.0156103)
Supplement: S6 Table — The 94 SNPs are ranked in increasing order of P-value and nominally significant associations are highlighted in bold. (DOCX) [file pone.0156103.s008.docx]

**S6 Table. Association results of the PSP GWAS technical validation phase.** The 94 SNPs are ranked in increasing order of *P*-value and nominally significant associations are highlighted in bold.

| **SNP** | **Chr.** | **Gene** | **Allele** | **HWE *P*** | **Case freq.** | **Control freq.** | ***P*** |
| --- | --- | --- | --- | --- | --- | --- | --- |
| rs10222715 | 4 | *-* | G | 0.848 | 0.554 | 0.337 | **1.10E-05** |
| rs488940 | 11 | *-* | A | 0.813 | 0.451 | 0.250 | **2.27E-05** |
| rs287903 | 6 | *ARID1B* | A | 0.023 | 0.891 | 0.734 | **2.31E-05** |
| rs2058487 | 9 | *-* | G | 0.246 | 0.359 | 0.184 | **5.55E-05** |
| rs10491529 | 9 | *-* | G | 0.404 | 0.370 | 0.194 | **5.91E-05** |
| rs1333199 | 10 | *-* | G | 1.000 | 0.315 | 0.151 | **6.35E-05** |
| rs9487033 | 6 | *-* | C | 0.474 | 0.630 | 0.438 | **1.14E-04** |
| rs17221652 | 10 | *ADARB2* | C | 0.003 | 0.462 | 0.291 | **1.18E-04** |
| rs11629958 | 15 | *SH3GL3* | C | 0.224 | 0.690 | 0.492 | **1.54E-04** |
| rs287916 | 6 | *ARID1B* | A | 0.041 | 0.880 | 0.742 | **1.74E-04** |
| rs230833 | 5 | *-* | A | 0.616 | 0.918 | 0.781 | **1.75E-04** |
| rs4397338 | 7 | *-* | A | 1.000 | 0.734 | 0.562 | **2.69E-04** |
| rs1397056 | 11 | *OR8U8* | T | 0.146 | 0.397 | 0.233 | **3.00E-04** |
| rs6983560 | 8 | *ZFAT* | G | 1.000 | 0.277 | 0.124 | **3.27E-04** |
| rs346501 | 4 | *ARHGAP24* | A | 0.255 | 0.538 | 0.355 | **3.52E-04** |
| rs6902892 | 6 | *CCDC162* | A | 0.082 | 0.663 | 0.492 | **3.71E-04** |
| rs7911954 | 10 | *-* | C | 0.401 | 0.342 | 0.194 | **3.92E-04** |
| rs4602358 | 3 | *CCK* | T | 0.376 | 0.625 | 0.450 | **5.47E-04** |
| rs487013 | 9 | *AKAP2* | G | 1.000 | 0.723 | 0.558 | **5.63E-04** |
| rs7831961 | 8 | *ZFAT* | C | 0.192 | 0.315 | 0.160 | **5.90E-04** |
| rs10956847 | 8 | *-* | C | 0.768 | 0.310 | 0.172 | **6.08E-04** |
| rs139167 | 22 | *PARVG* | G | 0.346 | 0.891 | 0.752 | **6.56E-04** |
| rs2962615 | 5 | *-* | C | 0.256 | 0.533 | 0.359 | **6.78E-04** |
| rs8055491 | 16 | *-* | A | 0.374 | 0.614 | 0.452 | **9.38E-04** |
| rs10820588 | 9 | *-* | T | 0.599 | 0.668 | 0.500 | **9.71E-04** |
| rs236715 | 20 | *-* | T | 0.815 | 0.880 | 0.750 | **1.03E-03** |
| rs17671063 | 7 | *-* | A | 0.718 | 0.761 | 0.605 | **1.04E-03** |
| rs459020 | 6 | *-* | G | 0.562 | 0.315 | 0.182 | **1.07E-03** |
| rs7374822 | 3 | *EPHB1* | A | 0.860 | 0.571 | 0.410 | **1.09E-03** |
| rs7682400 | 4 | *-* | T | 0.357 | 0.766 | 0.605 | **1.10E-03** |
| rs6531429 | 4 | *-* | T | 0.557 | 0.815 | 0.676 | **1.12E-03** |
| rs13188604 | 5 | *-* | G | 0.031 | 0.717 | 0.570 | **1.18E-03** |
| rs16855688 | 3 | *-* | A | 1.000 | 0.967 | 0.872 | **1.33E-03** |
| rs4883870 | 13 | *-* | G | 0.274 | 0.592 | 0.426 | **1.39E-03** |
| rs7741604 | 6 | *CDKAL1* | A | 0.238 | 0.940 | 0.824 | **1.40E-03** |
| rs12666340 | 7 | *C7orf58* | A | 0.280 | 0.598 | 0.437 | **1.49E-03** |
| rs4423896 | 4 | *-* | C | 0.539 | 0.462 | 0.310 | **1.90E-03** |
| rs752962 | 10 | *FRMD4A* | T | 0.565 | 0.799 | 0.655 | **1.92E-03** |
| rs1962137 | 11 | *-* | T | 0.392 | 0.853 | 0.718 | **1.96E-03** |
| rs4922683 | 11 | *LUZP2* | A | 0.043 | 0.902 | 0.777 | **1.97E-03** |
| rs4457905 | 14 | *PRKD1* | T | 0.595 | 0.332 | 0.198 | **1.97E-03** |
| rs9547912 | 13 | *-* | T | 0.834 | 0.451 | 0.301 | **1.99E-03** |
| rs1495980 | 6 | *-* | G | 0.780 | 0.326 | 0.191 | **2.14E-03** |
| rs7241671 | 18 | *ZNF407* | A | 0.519 | 0.940 | 0.841 | **2.15E-03** |
| rs13331343 | 16 | *CDH13* | A | 0.589 | 0.658 | 0.508 | **2.34E-03** |
| rs12027334 | 1 | *TNNI3K* | A | 1.000 | 0.810 | 0.675 | **2.36E-03** |
| rs2545886 | 16 | *DNAH3* | A | 0.471 | 0.957 | 0.859 | **2.49E-03** |
| rs8048056 | 16 | *RBFOX1* | C | 1.000 | 0.364 | 0.225 | **2.64E-03** |
| rs7463038 | 8 | *TACC1* | G | 0.633 | 0.152 | 0.066 | **2.95E-03** |
| rs2122914 | 16 | *-* | C | 0.482 | 0.652 | 0.504 | **3.00E-03** |
| rs6945688 | 7 | *PDE1C* | A | 0.858 | 0.696 | 0.554 | **3.02E-03** |
| rs10942788 | 5 | *IQGAP2* | G | 0.726 | 0.614 | 0.461 | **3.08E-03** |
| rs3097903 | 4 | *LOC285419* | A | 0.480 | 0.632 | 0.492 | **3.15E-03** |
| rs10903913 | 10 | *-* | A | 0.241 | 0.913 | 0.816 | **3.38E-03** |
| rs516081 | 9 | *-* | T | 1.000 | 0.451 | 0.309 | **3.47E-03** |
| rs3789950 | 10 | *TLL2* | T | 0.232 | 0.451 | 0.318 | **3.51E-03** |
| rs7767391 | 6 | *CDKAL1* | T | 0.824 | 0.859 | 0.734 | **3.55E-03** |
| rs2101167 | 3 | *LEKR1* | A | 0.630 | 0.978 | 0.898 | **3.94E-03** |
| rs10808167 | 7 | *MAGI2* | A | 0.697 | 0.522 | 0.383 | **4.05E-03** |
| rs4733649 | 8 | *-* | C | 0.291 | 0.429 | 0.301 | **4.07E-03** |
| rs4727161 | 7 | *-* | T | 0.337 | 0.780 | 0.651 | **4.34E-03** |
| rs4434965 | 11 | *-* | T | 1.000 | 0.495 | 0.357 | **4.59E-03** |
| rs11708202 | 3 | *SLC6A1* | G | 0.688 | 0.217 | 0.112 | **5.13E-03** |
| rs6466365 | 7 | *IMMP2L* | T | 0.853 | 0.712 | 0.582 | **5.78E-03** |
| rs922799 | 8 | *CSMD1* | C | 0.152 | 0.630 | 0.492 | **6.21E-03** |
| rs10825483 | 10 | *PCDH15* | A | 0.598 | 0.696 | 0.558 | **6.26E-03** |
| rs723436 | 13 | *-* | T | 0.283 | 0.679 | 0.555 | **6.68E-03** |
| rs1131535 | 3 | *TNFSF10* | T | 0.186 | 0.500 | 0.368 | **7.03E-03** |
| rs2041301 | 7 | *AAA1* | A | 0.378 | 0.603 | 0.465 | **7.14E-03** |
| rs10088760 | 8 | *-* | A | 0.219 | 0.663 | 0.535 | **7.20E-03** |
| rs4602638 | 5 | *-* | A | 0.270 | 0.717 | 0.594 | **7.21E-03** |
| rs2164512 | 16 | *-* | G | 0.757 | 0.275 | 0.168 | **7.34E-03** |
| rs155681 | 12 | *TMEM132D* | T | 0.053 | 0.598 | 0.461 | **8.20E-03** |
| rs10484048 | 14 | *-* | C | 0.741 | 0.924 | 0.837 | **9.14E-03** |
| rs612389 | 11 | *DLG2* | T | 0.794 | 0.880 | 0.787 | **1.10E-02** |
| rs6881724 | 5 | *-* | A | 0.833 | 0.826 | 0.723 | **1.15E-02** |
| rs12792701 | 11 | *-* | T | 0.251 | 0.913 | 0.824 | **1.20E-02** |
| rs11847260 | 14 | *-* | G | 0.714 | 0.565 | 0.445 | **1.24E-02** |
| rs6110533 | 20 | *MACROD2* | C | 0.279 | 0.679 | 0.562 | **1.30E-02** |
| rs9656970 | 8 | *-* | C | 0.500 | 0.370 | 0.264 | **1.51E-02** |
| rs1353318 | 8 | *-* | A | 0.527 | 0.913 | 0.829 | **1.71E-02** |
| rs17491067 | 14 | *NPAS3* | A | 0.608 | 0.326 | 0.223 | **1.85E-02** |
| rs10242076 | 7 | *MIR548I4* | T | 0.820 | 0.348 | 0.250 | **2.62E-02** |
| rs436563 | 9 | *PTPRD* | G | 1.000 | 0.473 | 0.370 | **3.50E-02** |
| rs8083684 | 18 | *-* | T | 1.000 | 0.967 | 0.917 | **3.94E-02** |
| rs1445324 | 11 | *-* | C | 0.699 | 0.446 | 0.349 | **4.39E-02** |
| rs12583307 | 13 | *-* | A | 0.703 | 0.734 | 0.646 | **4.98E-02** |
| rs2413151 | 22 | *SYN3* | C | 0.438 | 0.299 | 0.217 | 5.81E-02 |
| rs9423526 | 10 | *-* | T | 0.045 | 0.674 | 0.579 | 6.14E-02 |
| rs2926721 | 5 | *-* | A | 0.182 | 0.348 | 0.267 | 8.60E-02 |
| rs411167 | 9 | *-* | C | 0.135 | 0.348 | 0.271 | 1.05E-01 |
| rs9522832 | 13 | *-* | T | 0.478 | 0.560 | 0.504 | 2.41E-01 |
| rs10508279 | 10 | *-* | G | 0.921 | 1.000 | 0.968 | 9.85E-01 |
| rs10966315 | 9 | *-* | T | 0.921 | 1.000 | 0.988 | 9.85E-01 |

Abbreviations - Chr.: Chromosome; HWE *P*: Hardy Weinberg equilibrium *P*-value in controls; freq.: associated allele frequency; *P*: logistic regression *P*-value using the log-additive model.
